# Genetic and morphological characterization of the freshwater mussel clubshell species complex (*Pleurobema clava* and *Pleurobema oviforme*) to inform conservation planning

**DOI:** 10.1002/ece3.8219

**Published:** 2021-10-20

**Authors:** Cheryl L. Morrison, Nathan A. Johnson, Jess W. Jones, Michael S. Eackles, Aaron W. Aunins, Daniel B. Fitzgerald, Eric M. Hallerman, Tim L. King

**Affiliations:** ^1^ U.S. Geological Survey, Eastern Ecological Science Center, Leetown Research Laboratory Kearneysville West Virginia USA; ^2^ U.S. Geological Survey, Wetland and Aquatic Research Center Gainesville Florida USA; ^3^ U.S. Fish and Wildlife Service, Department of Fish and Wildlife Conservation, Virginia Polytechnic Institute and State University Blacksburg Virginia USA; ^4^ Department of Fish and Wildlife Conservation Virginia Polytechnic Institute and State University Blacksburg Virginia USA

**Keywords:** *COI*, cryptic biodiversity, endangered species, microsatellite DNA, mitochondrial DNA, morphometrics, NDI

## Abstract

The shell morphologies of the freshwater mussel species *Pleurobema clava* (federally endangered) and *Pleurobema oviforme* (species of concern) are similar, causing considerable taxonomic confusion between the two species over the last 100 years. While *P. clava* was historically widespread throughout the Ohio River basin and tributaries to the lower Laurentian Great Lakes, *P. oviforme* was confined to the Tennessee and the upper Cumberland River basins. We used two mitochondrial DNA (mtDNA) genes, 13 novel nuclear DNA microsatellite markers, and shell morphometrics to help resolve this taxonomic confusion. Evidence for a single species was apparent in phylogenetic analyses of each mtDNA gene, revealing monophyletic relationships with minimal differentiation and shared haplotypes. Analyses of microsatellites showed significant genetic structuring, with four main genetic clusters detected, respectively, in the upper Ohio River basin, the lower Ohio River and Great Lakes, and upper Tennessee River basin, and a fourth genetic cluster, which included geographically intermediate populations in the Ohio and Tennessee river basins. While principal components analysis (PCA) of morphometric variables (i.e., length, height, width, and weight) showed significant differences in shell shape, only 3% of the variance in shell shape was explained by nominal species. Using Linear Discriminant and Random Forest (RF) analyses, correct classification rates for the two species' shell forms were 65.5% and 83.2%, respectively. Random Forest classification rates for some populations were higher; for example, for North Fork Holston (HOLS), it was >90%. While nuclear DNA and shell morphology indicate that the HOLS population is strongly differentiated, perhaps indicative of cryptic biodiversity, we consider the presence of a single widespread species the most likely biological scenario for many of the investigated populations based on our mtDNA dataset. However, additional sampling of *P. oviforme* populations at nuclear loci is needed to corroborate this finding.

## Graphical abstract

Relationships between two imperiled and morphologically similar freshwater mussel species, *Pleurobema clava* and *Pleurobema oviforme*, are re‐examined using mitochondrial DNA, morphometrics, and nuclear microsatellites. While phylogenetic analysis of mitochondrial DNA suggests a single, widespread species, one population was significantly divergent based upon microsatellites and morphometrics. Sampling of additional populations and analysis with additional nuclear loci are recommended to further define management units.

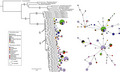

## 1 INTRODUCTION

A robust scientific basis for managing biodiversity originates with accurate delineation of species and population groups with independent evolutionary trajectories (Bernatchez & Wilson, 1998; De Queiroz, 2007; Pante et al., 2015; Vignieri et al., 2006). However, morphology‐based taxonomy does not always reflect evolutionary trajectories (Avise, 1994). While the most diverse in North America, native freshwater mussels (Bivalvia: Unionida) are among the most imperiled groups of animals (Haag, 2012; Lydeard et al., 2004; Williams et al., 1993). Many species inhabit small geographic ranges within stream networks and have limited (and often unknown) dispersal abilities due to reliance on specific host fishes for metamorphosis from larval (glochidia) to free‐living life stages (juvenile, sub‐adult, adult), making populations easily fragmented and particularly susceptible to habitat modification, pollution, and over‐harvest (Haag & Williams, 2014; Neves, 1993; Williams et al., 1993). Despite the recognition that many native mussels face high extinction and imperilment rates (Haag & Williams, 2014), knowledge of basic biology, ecology, and taxonomy for some unionids remains lacking, which limits the ability of natural resource managers to take immediate conservation action. Identifying discrete morphological characters to diagnose species or determine evolutionary lineages can be challenging due to similarity in overall shape and appearance. Further, conchological features are heritable, but may also be influenced by ecological factors (Agrell, 1948; Kodolova & Logvinenko, 1973; Ortmann, 1920; Watters, 1994) and can exhibit extensive morphological variation within and among river drainages (Graf, 1997, 1998; Hyde et al., 2020; Inoue et al., 2013, 2015; Johnson et al., 2018; Ortmann, 1920). Such phenotypic plasticity often hinders our ability to delimit species and design appropriate management plans (Burlakova et al., 2012; Inoue et al., 2013, 2014, 2018; Johnson et al., 2018).

Many species belonging to the North American Tribe Pleurobemini (Bivalvia: Unionida) exhibit substantial variation in shell morphology, which has led to taxonomic confusion and reliance on geography to delineate species boundaries. In fact, species belonging to the genus *Pleurobema* have been described as “the most perplexing group of unionids” in some basins (Williams et al., 2008), with 23 species currently recognized (Williams et al., 2017). Most *Pleurobema* species are threatened or endangered at either the state or national level, so difficulty delineating species using morphological characters has strong potential to affect conservation and management efforts (Shea et al., 2011).

The Clubshell, *Pleurobema clava* (Lamarck, 1819), is a freshwater bivalve that inhabits clean, coarse sand, and gravel runs in small‐ to moderate‐sized streams in the eastern United States. *Pleurobema clava* was historically widespread and abundant in the Ohio River basin and tributaries of western Lake Erie (Dean, 1890; Watters, 1988) yet has experienced a 95% range reduction after being found in only 13 of 100 historically known localities (U.S. Fish & Wildlife Service, 1994). *Pleurobema clava* was listed as an endangered species in 1993 (U.S. Fish and Wildlife Service, 1993) under the Endangered Species Act (ESA 1973, as amended). Little is known about the ecology of *P. clava*, or about the connectivity between the remaining, widely scattered populations, and no genetic information exists on population structure, levels of gene flow, or relatedness within or among populations of *P. clava*.

**FIGURE 1:**
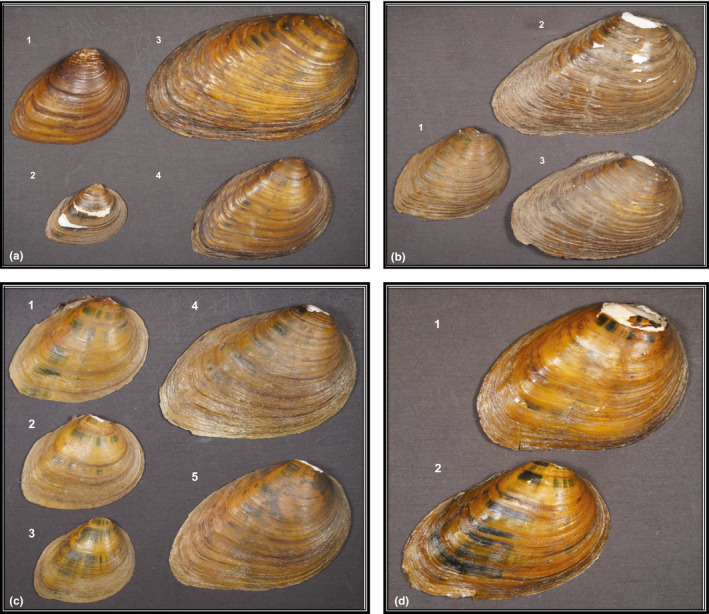
Photographs of individual valves (right only) of *Pleurobema clava* showing morphological variation among shells collected from locations throughout the species range in the Ohio River and Lake Erie watersheds of Ohio (OH), Pennsylvania (PA), and West Virginia (WV), U.S.A. Measurements are for length (L) of individuals in each panel. Panel a: Valves collected from Fish Creek, Maumee River, Lake Erie drainage, Williams County, OH, St. Joseph Township on July 28, 2010, east of CR‐18, where (1) *L* = 48 mm, (2) *L* = 32 mm, (3) *L* = 79 mm, and (4) *L* = 55 mm. McClung Museum Lot # 5143. Panel b: Valves collected from LeBoeuf Creek, Allegheny River drainage, Erie County, PA, on May 12, 1994, east of Route 19, 3 km south of LeBoeuf Gardens where (1) *L* = 49 mm (2) *L* = 84 mm, and (3) *L* = 71 mm. McClung Museum Lot # 0983. Panel c: Valves collected from the Allegheny River, Venango County, PA, President Township on September 26, 1995, at head of island at the mouth of Hemlock Creek, where (1) *L* = 46 mm, (2) *L* = 41 mm, (3) *L* = 38 mm, and (4) *L* = 63 mm, (5) *L* = 61 mm. McClung Museum Lot # 16447. Panel d: Valves collected from Elk River, Braxton County, WV, on May 11, 1970, east of Gassaway, 3 miles west of Sutton, Otter Township, where (1) *L* = 54 mm, and (2) *L* = 53 mm. McClung Museum Lot # 0978. All photographs taken by J.W. Jones

**FIGURE 2:**
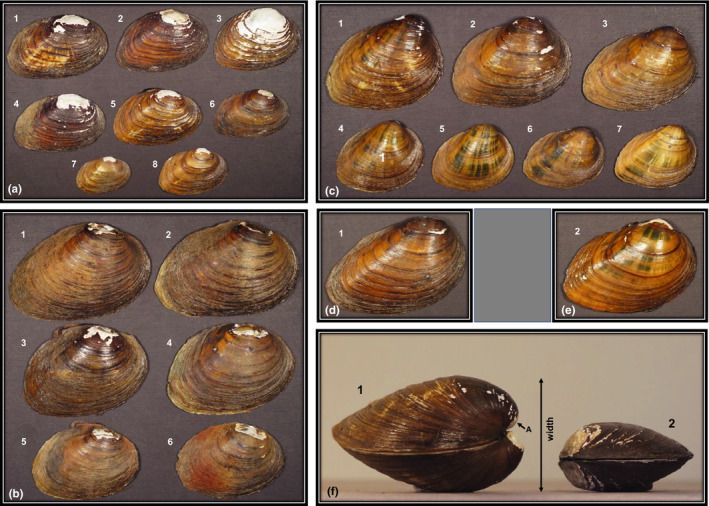
Photographs of individual valves (right only) of *Pleurobema oviforme* showing morphological variation among shells collected from locations throughout the species range in the upper and middle Tennessee River and Cumberland River watersheds of Alabama (AL), Kentucky (KY), Tennessee (TN) and Virginia (VA), U.S.A. Measurements are for length (L) of individuals in each panel. Panel a: Valves collected from the North Fork Holston River, Smyth County, VA, near Riverside on August 8, 1995, where (1) *L* = 65.5 mm, (2) *L* = 61.0 mm, (3) *L* = 59.5 mm, (4) *L* = 57 mm, (5) *L* = 58 mm, (6) *L* = 59.8 mm, (7) *L* = 45.7, and (8) *L* = 35.5. McClung Museum Lot # 15462. Panel b: Valves collected from the Hiwassee River, Polk County, TN, at the Route 68 Bridge on July 23, 2007, where (1) *L* = 53.9 mm, (2) *L* = 54 mm, (3) *L* = 48.2 mm, (4) *L* = 48.3 mm, (5) *L* = 40.8 mm, and (6) *L* = 40 mm. McClung Museum Lot #4317. Panel c: Valves collected from the Paint Rock River, Jackson County, AL, at Jones property (RM 34.7) on October 11, 2012, where (1) *L* = 61.5 mm, (2) *L* = 63 mm, (3) *L* = 60.3 mm, (4) *L* = 46.2 mm, (5) *L* = 42.3 mm, (6) *L* = 42 mm, and (7) Length = 41.7. McClung Museum Lot # 5318. Panel d: Valve collected from Buck Creek, Pulaski County, KY, Highway 70 Bridge on August 23, 1999, *L* = 66.5 mm, McClung Museum Lot #12561. Panel e: Valve collected from Red River, Robertson County, TN, Route 161, 2 miles south of Keysburg on August 27, 1977, *L* = 47.2 mm, McClung Museum Lot #16432. Panel f: Comparison of valve width of specimens collected from (1) Paint Rock River, *L* = 62 mm and Width = 33.6 mm (Specimen #1 from B), and (2) North Fork Holston River, Length = 61 mm and Width = 20 mm (Specimen #2 from A). Small arrow A = umbos of specimen 1 protrude past the hinge line, whereas umbos of specimen 2 do not. All photographs taken by J.W. Jones

The relationship and taxonomic status between *P. clava* and another currently recognized species, the Tennessee Clubshell, *P. oviforme* (Conrad, 1834), remains uncertain. Past taxonomic confusion between the two species likely centers on the morphological similarity between the majority of *P. oviforme* populations, and the congener *P. clava*. *Pleurobema clava* is elongate or “club‐like” (*clava* = “club” in Latin) in appearance (Figure 1a–d), with umbos set‐back anteriorly, inflated and extending 2–4 mm beyond the hinge line, whereas *P. oviforme* is oval or “egg‐like” (*oviforme* = “egg‐form” in Latin) in appearance, with umbos more centrally positioned and much less inflated, typically either even with the hinge line or barely extending beyond it (Figure 2a–f; Parmalee & Bogan, 1998; Watters et al., 2009; Williams et al., 2008). Specifically, shells of *P. oviforme* from the middle (e.g., Paint Rock River) and lower (e.g., Duck River) sections of the Tennessee River basin have inflated umbos that extend well beyond the hinge line, greater width, and tend to be more elongate and have an angular posterior ridge, similar to *P. clava* from the Great Lakes region and Ohio River system. In the Cumberland River basin, however, *P. oviforme* is thought to replace *P. clava* in headwater streams because shells tend to be more compressed and less elongate and have an angular posterior ridge, with umbos that are located anteriorly and extending beyond the hinge line (Figure 2d and e) (Haag & Cicerello, 2016; Parmalee & Bogan, 1998; Watters et al., 2009; Williams et al., 2008). However, when shells of *P. clava* are compared visually to shells of *P. oviforme* from the upper Tennessee River basin, more pronounced differences in shape are evident. The shells of *P. oviforme* from the upper Tennessee River basin are more flattened or compressed (Figure 2f), and the posterior ridge is more rounded and not sharp or angular. These morphological differences allow easy identification of specimens obtained from distant regions of each species distribution, but in the adjacent regions of their distributions, the morphological similarities and intergradation between the two shell forms remain a source of taxonomic confusion.

The similarities in shell morphologies and disjunct distributions have led some to hypothesize that *P. clava* and *P. oviforme* may encompass either sibling species (USFWS, 1994) or clinal variation of a single species (Ortmann, 1925; Williams et al., 2008), while others have hypothesized that undescribed or cryptic diversity may exist within the species complex (Haag & Cicerello, 2016; Schilling, 2015). Based on shell morphology, malacologists consider the historical distribution of *P. oviforme* to be confined to the Tennessee and Upper Cumberland River basins, while *P. clava* is widespread throughout the Ohio River basin. *Pleurobema clava* presently occurs in rivers in six states (Michigan, Ohio, Indiana, West Virginia, Kentucky, and Pennsylvania), with large extant populations occurring in the Tippecanoe River, Indiana (Cummings & Berlocher, 1990; Cummings et al., 1992) and in the Allegheny River (Pennsylvania) and its tributaries (USFWS, 1994). *Pleurobema oviforme* is considered extant in the middle and upper Tennessee River drainages, including populations in the Clinch, Duck, Hiawassee, North Fork Holston, Paint Rock, Tellico, Pigeon and other rivers, including numerous smaller tributaries to these streams. Additionally, a few small populations are extant in the upper Cumberland River drainage (Haag & Cicerello, 2016; Williams et al., 2008). Although *P. oviforme* remains widespread geographically, the species has lost 58–76% of its former range (Fitzgerald et al., 2021).

Taxonomic assessments have generally recognized *P. clava* and *P. oviforme* as valid species (Turgeon et al., 1998; Williams et al., 1993, 2017). However, molecular studies utilizing mtDNA have indicated that *P. clava* and *P. oviforme* are closely related (Campbell & Lydeard, 2012; Campbell et al., 2005, 2008), and most recently, molecular evidence for the conspecific status of these species was presented in a comprehensive phylogenetic analysis of the tribe Pleurobemini (Inoue et al., 2018). Unlike *P. clava*, *P. oviforme* is not currently protected under the ESA but is being considered for listing (Federal Register 76[187]:59836–59862); therefore, determining the relationship and resolving taxonomic uncertainties between these taxa has important implications for conservation efforts and decision making regarding listing and protection.

In this study, we analyze morphological and genetic (mitochondrial and nuclear DNA) variation from samples collected throughout the ranges of both *P. clava* and *P. oviforme* to test species boundaries and characterize genetic population structure. First, we assessed genetic distinctiveness by testing monophyly of each putative species expected under the Phylogenetic Species Concept (PSC, Eldridge & Cracraft, 1980; Nelson & Platnick, 1981) using two mitochondrial genes (*COI* and *ND1*). In addition, we used morphometric data collected from museum specimens identified as *P. clava* and *P. oviforme* to test whether the two species can be reliably distinguished based on morphological characters. We then used 13 novel nuclear microsatellite markers to further evaluate species boundaries and determine whether population structuring exists across the broad geographic range of the species complex. We discuss important limitations in our genetic and morphological datasets for delineating these taxa, and additional sampling that is needed to refine our understanding of the two species and potential management units (MUs). These findings have important taxonomic and management implications given the conservation status of *P. clava* and the imminent ESA listing decision for *P. oviforme*.

## 2 MATERIALS AND METHODS

### 2.1 Taxon sampling and DNA extraction

**FIGURE 3:**
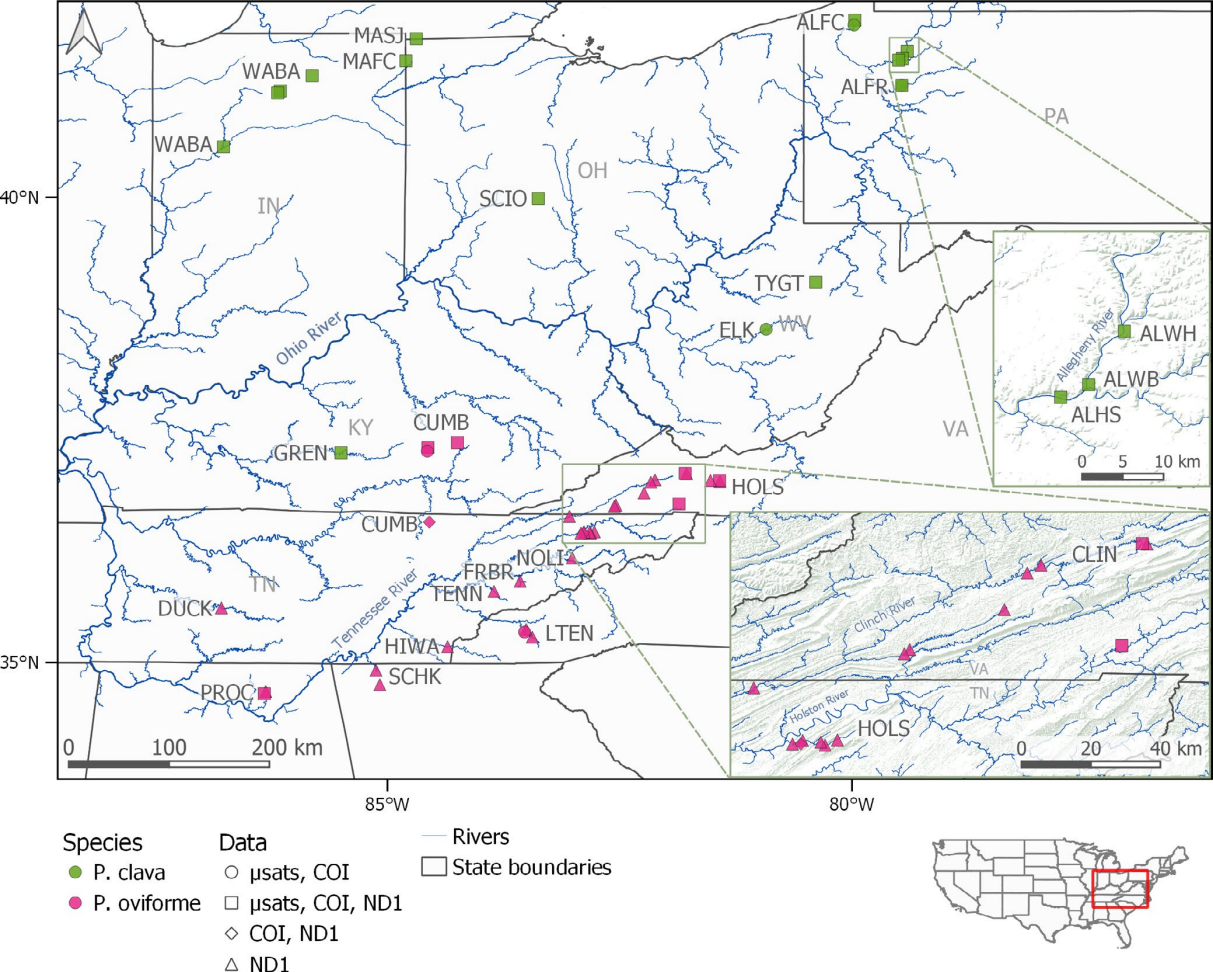
Sampling locations for *Pleurobema clava* (green) and *Pleurobema oviforme* (red) samples analyzed in this study, with shapes indicating inclusion in different datasets. Site codes are given in Table 1

Non‐lethal tissue samples (mantle clips following Berg et al., 1995 or swabs following Henley et al., 2006) were taken for DNA analyses from individuals of *P. clava* and *P. oviforme* throughout their present ranges in three watersheds (Great Lakes, Ohio, and Tennessee), including 18 drainages in 11 states (Table 1, Figure 3). Due to uncertainty with field identification and differences in sample sizes, individuals were grouped by collection location within drainages (e.g., Allegheny River for *COI* and microsatellites) or by drainage (*ND1*) and will be referred to as “populations” herein (see Table 1 for details). Collection details, museum catalog numbers, and GenBank accession numbers are provided in Morrison et al. (2021).

**TABLE 1:** Sampling sites, site codes, and sample sizes (*N*) for mitochondrial DNA sequences (*COI* and *ND1*) and nuclear microsatellite loci (µsats) for *Pleurobema clava* and *Pleurobema oviforme* included in this study

Taxa	Site	River	State	Drainage	Site code	*N* *COI*	*N* *ND1*	*N* µsats
*P. clava*	Big Meadow	St. Joseph	MI	Maumee (Lake Erie)	MASJ	13	8	14
*P. clava*	Fish Creek	Fish Creek	OH	Maumee (Lake Erie)	MAFC	3	4	3
*P. clava*	West Hickory	Allegheny	PA	Allegheny	ALWH	31	9	33
*P. clava*	Hunter Station	Allegheny	PA	Allegheny	ALHS	32	10	32
*P. clava*	Walnut Bend	Allegheny	PA	Allegheny	ALWB	30	6	35
*P. clava*	Franklin	Allegheny	PA	Allegheny	ALFR	38	1	42
*P. clava*	French Creek	Allegheny	PA	Allegheny	ALFC	8	2	8
*P. clava*	Mill Creek	Elk	WV	Elk	ELK	8	2	7
*P. clava*	Hackers Creek	Hackers Creek	WV	Tygart	TYGT	2	1	5
*P. clava*	Tippecanoe	Tippecanoe	IN	Wabash	WABA^a^	12	10	17
*P. clava*	Little Darby	Little Darby	OH	Scioto	SCIO	3	3	3
*P. clava*	Greensburg	Green	KY	Green	GREN	3	1	5
*P. oviforme*	Buck/Roundstone Creek	Cumberland	KY	Cumberland	CUMB^a^	7	5	4
*P. oviforme*	Indian Creek	Clinch	VA	Clinch	CLIN	1	17	1
*P. oviforme*	Paint Rock	Paint Rock	AL	Paint Rock	PROC	2	3	1
*P. oviforme*	L. Tennessee^b^	L. Tennessee^b^	NC	L. Tennessee^b^	LTEN^a^	3	13	1
*P. oviforme*	NF Holston^c^	NF Holston^c^	VA	Holston	HOLS	28	59	24
*P. oviforme*	Lillard Mill	Duck	TN	Duck	DUCK	0	3	0
*P. oviforme*	Sevierville	Little Pigeon	TN	French Broad	FRBR	0	3	0
*P. oviforme*	Turtletown	Hiwassee	TN	Hiwassee	HIWA	0	5	0
*P. oviforme*	Coulter's Bridge	Little River	TN	Tennessee	TENN	0	2	0
*P. oviforme*	Sinking Spring	Little Chucky	TN	Nolichucky	NOLI	0	3	0
*P. oviforme*	Ringgold^a^	S Chickamauga	GA	S Chickamauga	SCHK^a^	0	8	0
Total						224	178	235

Note

For microsatellite analyses, the single samples from the Clinch, Paint Rock, and Little Tennessee were combined as they all originated from the Tennessee watershed (TENN).

^a^ Includes multiple sampling sites, see Morrison et al. (2021) for details.

^b^ Little Tennessee.

^c^ North Fork Holston.

Genomic DNA was isolated from DNA swabs or mantle tissue preserved in 95% EtOH using the Puregene DNA Extraction Kit (Gentra Systems, Inc.) and resuspended in TE buffer (10 mM Tris‐HCl, pH 8.0, 1 mM EDTA). DNA concentrations were determined by fluorescence assay (Labarca & Paigen, 1980), and integrity of the DNA was visualized on 1% agarose gels (Sambrook et al., 1989).

### 2.2 Mitochondrial DNA sequencing and analyses

We sequenced two mitochondrial DNA (mtDNA) protein‐encoding genes for phylogenetic and phylogeographic analyses: the *cytochrome oxidase subunit I* (*COI*) and the *NADH dehydrogenase subunit I* (*ND1*) genes. Mitochondrial genes were amplified from genomic DNA using the polymerase chain reaction (PCR), with *COI* primers (dgLCO‐1490 and dgHCO‐2198) and thermal cycling conditions of Folmer et al. (1994) and *ND1* primers (Leu‐uurF and LoGlyR) and conditions of Serb et al. (2003). Amplification reactions for both *COI* and *ND1* were performed in 20‐μl reactions that consisted of the following components and final concentrations: 1 X PCR Buffer (Promega, Madison, WI), 3.75 mM MgCl_2_, 0.3 of each dNTP, 0.2 mM of each primer (from 5 mM stock), 0.5% Tween‐20, 0.08 units/μl of *Taq* polymerase (Promega), and 10 ng/ul template DNA. Visual inspection for the targeted amplification product was confirmed on 1% agarose gels.

Polymerase chain reaction products from mtDNA genes were purified with Exonuclease I and Shrimp Alkaline Phosphatase (Promega Corp.) and then used as templates in sequencing reactions with the ABI Prism BigDye Terminator Cycle Sequencing Ready Reaction Kit (Applied Biosystems) using the amplification primers. Sequencing reactions were run using a PTC‐200 Thermal Cycler (MJ Research) and subjected to electrophoresis on an ABI Prism 3130™ Genetic Analyzer. Products of forward and reverse sequencing reactions were assembled, forming a consensus sequence for each individual and gene using Sequencher 5.4 (GeneCodes Corporation). Multiple alignments were performed for each gene region using Geneious software v. 11.1.5 (https://www.geneious.com) with default settings in the Geneious alignment algorithm. The mtDNA alignments were translated into amino acids to ensure the absence of stop codons and indels. Additional sequences (*COI* = 5, *NDI* = 106) were downloaded from GenBank for phylogenetic and phylogeographic analyses (see Morrison et al., 2021 for details).

Population diversity indices, such as the number of segregating sites (*S*), number of haplotypes (*h*), haplotype diversity (*H_d_*), and nucleotide diversity (*π*), were calculated per population and gene region using DnaSP version 6.12.03x64 (Rozas et al., 2017). Neutrality indices (Tajima's *D* (Tajima, 1989) and Fu's *Fs* (Fu, 1997)) were calculated for each population and gene region. The average (*D_xy_*) and net (*D_a_*) number of nucleotide substitutions per site were calculated between populations for each gene region using DnaSP. Genetic differentiation among populations was estimated by the haplotype‐based method (*H_s_*; Hudson et al., 1992), with significance tested via a chi‐square table in DnaSP, along with gene flow estimates (*F*_st_ and number of migrants, *N_m_*; Hudson et al., 1992). Phylogenetic maximum‐likelihood (ML) and maximum parsimony (MP) analyses were performed separately for each mtDNA gene. Nucleotide substitution models were determined for six partitions in ModelFinder (Kalyaanamoorthy et al., 2017) for ML phylogenetic analyses: *COI* 1st position—TN+F+I; *COI* 2nd position—F81+F; *COI* 3rd position—TPM3u+F; *ND1* 1st position—TNe+I; *ND1* 2nd position—HKY+F; and *ND1* 3rd position—TN+F. ML analyses were performed using IQ‐TREE v 2.0.6 (Nguyen et al., 2015) with 10 independent runs of initial tree search and 10,000 ultrafast bootstrap replicates (BS) to assess nodal support (Hoang, Chernomor, et al., 2018). MP analyses were performed with default parameters and 1000 ultrafast bootstrap replicates using MPBoot (Hoang, Vinh, et al., 2018). Resulting phylogenies for each mtDNA gene region were reported as 50% majority‐rule consensus trees. Haplotype networks were constructed based upon mitochondrial *COI* and *ND1* data separately using the TCS method (Clement et al., 2000) in PopArt v1.7 (Leigh & Bryant, 2015), which follows the parsimony‐based approach of Templeton et al. (1992, “TCS” method, after the authors).

### 2.3 Morphometrics

We used museum specimens (McClung Museum of Natural History and Culture—Knoxville, TN, and Florida Museum of Natural History—Gainesville, FL) identified as *P. clava* and *P. oviforme* to evaluate morphological variation throughout the range of both species. A single valve from each individual was positioned with ventral margins of anterior and posterior adductor muscle scars on a horizontal plane before taking the measurements to the nearest 0.01 mm using digital calipers. Total length anterior to posterior (length) parallel to the adductor scar plane, width at interdentum (width), and height from intersection of hinge ligament and umbo to the ventral margin of the shell (height) parallel to the adductor scar plane were measured. Valves were weighed to the nearest 0.1 g using a digital scale.

We used log_e_‐transformed measurement values to produce a scale‐invariant matrix while preserving information about allometry (Kowalewski et al., 1997; Strauss, 1985). We used the three log_e_‐transformed variables to calculate three ratios: height/length, width/length, and width/height. We used principal components analysis (PCA) and random forest analysis (RF) applied to standardized ratios to examine morphological variation among species. The random forest analysis was conducted using 80% (238 individuals) of the data as the training dataset and 20% (60 individuals) of the data as the validating dataset. After plotting the out‐of‐bag (OOB) error rate and observing how it declined and stabilized, a total of 500 trees was used for the analysis. The random forest analysis was conducted using the “rattle” package (Williams, 2011) as implemented in program R (R Core Team, 2019). To further evaluate the ability of morphological measurements to correctly classify individual shells into each respective species category, we conducted a linear discriminant analysis (LDA) with cross‐validation on data rarified to the lowest sample size (*N* = 129) using the lda function in the MASS package (Venables & Ripley, 2002) in R v. 4.0.2 (R Core Team, 2019).

### 2.4 Microsatellite genotyping and population genetic analyses

Two microsatellite‐enriched libraries were prepared by Genetic Identification Services, Inc. (GIS, http://www.genetic‐id‐services.com/), using magnetic bead capture technology (Peacock et al., 2002) and tetranucleotide microsatellite motif repeat capture molecules TACA and TAGA. Three hundred clones that contained repeats of appropriate length (>10 repeats) and had adequate flanking regions were targeted for primer development, including a modified 19‐bp M13 tag on the 5′ end of each forward primer (Boutin‐Ganache et al., 2001). PCR conditions were optimized as described in King et al. (2006), using genomic DNA from four individuals of *P. clava* from the Allegheny French Creek (ALFC), Maumee St. Joseph (MASJ), and Fish Creek (MAFC) populations plus four *P. oviforme* from the Cumberland drainage.

Based on size, strength of PCR amplification, ease of scoring, and observed heterozygosity, 13 loci were labeled utilizing the ABI Prism™ 5‐dye filter set (FAM, VIC, NED, PET, and LIZ). Loci were amplified individually, and PCR products were multiplexed into four size‐ and dye‐compatible groups. Microsatellite DNA amplification reactions consisted of 1 X PCR Buffer (Promega), 3.75 mM MgCl_2_, 0.3 of each dNTP, 0.2 mM of each primer (from 5 mM stock), 0.5% Tween‐20, 0.08 units/μl of *Taq* polymerase (Promega), and 20 ng/μl template DNA in a volume of 10 μl. Amplifications were carried out using the following thermal cycler profile: initial denaturing at 94°C for 2 min; 35 cycles of 94°C C for 40 s, 58°C C for 40 sec, 72°C C for 1 min; and a final extension at 72°C C for 5 min. Amplified, labeled PCR products were subjected to capillary electrophoresis on an ABI Prism™ 3130 XL Genetic Analyzer (Applied Biosystems, Inc.) with Gene Scan 500 LIZ size standards. GeneScan 3.7 analysis software, Genotyper 4.0 (Applied Biosystems), and GeneMapper (ver. 4) (Thermo Fisher Scientific) were used to score, bin, and output allelic (and genotypic) data following the protocols described in King et al. (2001).

The quality of microsatellite genotyping was checked by several measures. First, duplicate multi‐locus genotypes (MLGs) were located using GenAlEx v. 6.5 (Peakall & Smouse, 2006). MICROCHECKER v. 2.2 (Van Oosterhout et al., 2004) was used to check for genotyping errors, large allele dropout, and segregation of null alleles. Fisher's exact test was used to test for linkage disequilibrium (LD), and exact tests for Hardy–Weinberg equilibrium (HWE) were performed in GENEPOP on the Web (Raymond & Rousset, 1995). Sequential Bonferroni adjustments were applied for multiple tests (Rice, 1989). The power to distinguish unique MLGs was calculated as the probability of identity (*P*_ID_; Peakall & Smouse, 2006) in GenAlEx. Since sample sizes varied among populations, allelic richness, or the number of alleles per population, was calculated by rarefaction (Kalinowski, 2004) to compensate for unequal sample sizes using HP‐Rare (Kalinowski, 2005). Kinship coefficients were calculated among all individuals in Genodive v. 3.04 (Meirmans, 2020).

Traditional *F*‐statistics (Wright, 1951) were used to test for population structuring within and among drainages and watersheds for microsatellite data using analysis of molecular variance (AMOVA, Excoffier et al., 1992; Michalakis & Excoffier, 1996) in GenAlEx. To examine pairwise population structuring, we calculated two complementary measures in Genodive: the fixation index *G*′_st_ (Nei, 1987) and Jost's *D* (Jost, 2008), assessing significance with 9,999 permutations. Relationships among populations were assessed via the neighbor‐joining method (Saitou & Nei, 1987) using Nei's standard genetic distances (*D_A_*, Nei, 1972) in Poptree2 (Takezaki et al., 2010). Population‐based microsatellite analyses included grouping of single samples from the Little Tennessee (LTEN), Paint Rock (PROC), and Clinch (CLIN) collections into a Tennessee (TENN) population (Table 1).

Two different clustering methods were utilized to explore potential structuring within the MLG dataset. First, a Bayesian model‐based clustering approach was implemented in STRUCTURE v. 2.4 (Pritchard et al., 2000) to determine the number of clusters (*K*) within the MLG dataset by minimizing deviation from HWE within clusters. Values of *K* = 1 to 15 were tested, where *K* = 1 represents a single panmictic population and *K* = 15 represents the 15 populations that were sampled in this study. Twenty replicate simulations were run with 200,000 Markov chain Monte Carlo repetitions and a burn‐in of 100,000 with an admixture model, assuming independent allele frequencies across populations as priors (Hubitz et al., 2009). Results from replicate iterations were summarized using default settings in CLUMPAK (Kopelman et al., 2015) implemented in StructureSelector (Li & Liu, 2018); where the Evanno method (∆*K*, Evanno et al., 2005) and the posterior probability (PP) of each *K*‐value across replicates were calculated following Bayes' rule (p. 13, Pritchard & Wen, 2004) noted as ln Pr(*X*|*K*) (Pritchard et al., 2000) and were used to discern the best‐supported number of clusters using both the maximum‐likelihood scores and Δ*K* methods. Second, a discriminant analysis of principal components (DAPC; Jombart et al., 2010) analysis was performed in the R package adegenet (Jombart, 2008). Unlike the STRUCTURE analysis, the DAPC analysis clusters genetically similar individuals in multivariate space based on allelic composition without reliance on HWE and LD assumptions. Genetic isolation by distance (IBD) was analyzed using a Mantel test (Mantel, 1967) comparing pairwise genetic distances among populations (Nei's *D_A_*) with geographic river distances in adegenet. River distances were calculated as the shortest path between populations along U.S. National Hydrography flowlines (NHDPlus version 2) referenced to the Albers Equal Area North American Datum 1983 Coordinate Reference System (EPSG = 42303) using the packages sf and stplanr (Lovelace & Ellison, 2018; Pebesma, 2018; R Core Team, 2019).

The program BOTTLENECK v 1.2.02 (Cornuet & Luikart, 1996) was used to determine whether any populations experienced a recent reduction in population size, or population bottleneck, which would produce evidence of heterozygote excess (*Hx*; Cornuet & Luikart, 1996; Luikart et al., 1998). Two mutation models were calculated, including the conservative stepwise mutation model (SMM) and the two‐phase model (TPM) allowing for both stepwise and multi‐step mutations, as assumed to be reasonable for most microsatellites (Di Rienzo et al., 1994). For the TPM model, a variance of 12 was used (Piry et al., 1999) and the proportion of stepwise mutations in the model was set to 90 (Dussex et al., 2015; Garza & Williamson, 2001). Significance of heterozygote excess was tested using the sign test (Cornuet & Luikart, 1996), the Wilcoxon signed‐rank test (Luikart & Cornuet, 1998), and the mode‐shift indicator (Luikart et al., 1998) based on the presence of a mode shift in the distribution of allele frequencies.

## 3 RESULTS

### 3.1 Mitochondrial DNA

We generated 219 *COI* and 70 *ND1* DNA sequences for this study (Table 1). All novel sequences generated from this study are accessible on GenBank (*COI*: MT991776–MT991982; *ND1*: MW005982–MW006051). The *P. clava*/*oviforme COI* sequence alignment of 224 sequences was 572 bp in length, including 29 variable sites and nucleotide diversity *π* = 0.0061. The *ND1* alignment of 176 sequences was trimmed to 775 bp in length to minimize missing data, contained 55 variable sites, and nucleotide diversity *π* = 0.0049. No gaps or stop codons were detected in either protein‐encoding gene sequence.

Summary statistics for nucleotide and haplotype diversity for the *COI* and *ND1 P. clava*/*oviforme* datasets are shown in Table 2. The number of segregating sites (*S*) was higher for *ND1* (55) than for *COI* (29). Similarly, haplotype diversity (*H_d_*), which accounts for different sample sizes, was higher for the *ND1* compared with *COI* datasets (0.933 vs. 0.564, respectively). Haplotype diversity (*H_d_*) varied substantially among populations for *COI*, ranging from 0 in the Tygart (TYGT), Scioto (SCIO), and LTEN populations to 0.8228 in the HOLS and was consistently low in the five Allegheny (ALLE) and Elk (ELK; Ohio drainage) populations (0.131–0.25; Table 2), whereas *H_d_* was high for most populations at *ND1* (0.62–1), with the exception of St. Joseph/Maumee ([MASJ], 0.4103; Table 2).

**TABLE 2:** Summary of DNA sequence variation at the mitochondrial *COI* and *ND1* genes for *Pleurobema clava* and *Pleurobema oviforme*, where *n* is the number of sequences, *S* is the number of segregating sites, *h* is the number of haplotypes (number of haplotypes unique to population), *H_d_* is haplotype diversity, *k* is the number of pairwise differences, and *π* is the nucleotide diversity

Gene	Taxon	Site	*n*	*S*	*h*	*H_d_*	*k*	*π*
*COI* (572 bp)	*P. clava*	MASJ	13	9	3 (0)	0.4103	2.4103	0.0042
	*P. clava*	MAFC	3	3	2 (0)	0.6667	2	0.0035
	*P. clava*	ALHS	32	9	3 (0)	0.2319	1.869	0.0033
	*P. clava*	ALWH	31	10	4 (0)	0.4323	1.8495	0.0033
	*P. clava*	ALWB	30	6	3 (0)	0.131	0.4	0.0007
	*P. clava*	ALFR	38	15	4 (1)	0.2006	0.8393	0.0015
	*P. clava*	ALFC	8	9	2 (1)	0.25	2.25	0.004
	*P. clava*	ELK	8	9	2 (0)	0.25	2.25	0.004
	*P. clava*	TYGT	2	0	1 (0)	0	0	0
	*P. clava*	WABA^1^	12	10	3 (1)	0.3182	1.6667	0.0029
	*P. clava*	SCIO	3	0	1 (0)	0	0	0
	*P. clava*	GREN	3	2	2 (0)	0.6667	1.3333	0.0023
	*P. oviforme*	CUMB^1^	7	2	2 (1)	0.476	0.952	0.002
	*P. oviforme*	HOLS	28	15	8 (5)	0.8228	4.3942	0.0077
	*P. oviforme*	LTEN	3	5	2 (2)	0.667	3.333	0.0058
	*P. oviforme*	PROC	2	4	2 (1)	1	4.000	0.0070
	*P. oviforme*	CLIN	1	NA	1 (1)	NA	NA	NA
Average						0.564	3.448	0.0061
Total			224	29	20 (13)			
*ND1* (775 bp)	*P. clava*	MASJ	8	6	3 (0)	0.4643	1.8571	0.0027
	*P. clava*	MAFC	4	5	3 (0)	0.8333	3.1667	0.0046
	*P. clava*	ALLE^1^	28	11	8 (2)	0.6217	1.7355	0.0025
	*P. clava*	ELK	2	6	2 (0)	1	6	0.0077
	*P. clava*	WABA^1^	10	8	6 (1)	0.9111	2.8889	0.0042
	*P. clava*	TYGT	1	NA	1 (0)	NA	NA	NA
	*P. clava*	SCIO	3	3	3 (0)	1	2	0.0028
	*P. clava*	GREN	1	NA	1 (0)	NA	NA	NA
	*P. oviforme*	CUMB^1^	5	7	3 (3)	0.8333	3.5	0.0051
	*P. oviforme*	CLIN^1^	17	12	8 (6)	0.8529	2.7353	0.004
	*P. oviforme*	PROC	3	4	2 (1)	0.6667	2.6667	0.0039
	*P. oviforme*	HOLS^1^	59	18	15 (7)	0.886	2.9515	0.0043
	*P. oviforme*	LTEN	13	6	3 (1)	0.7	2.8	0.0041
	*P. oviforme*	DUCK	3	0	1 (0)	0	0	0
	*P. oviforme*	FRBR	3	7	3 (1)	1	4.6667	0.0068
	*P. oviforme*	HIWA	5	8	4 (3)	0.9	4.4	0.0064
	*P. oviforme*	TENN	2	4	2 (2)	1	4	0.0059
	*P. oviforme*	NOLI	3	6	3 (1)	1	4	0.0059
	*P. oviforme*	SCHK^1^	8	6	6 (4)	0.8	2.4667	0.0036
Average						0.933	3.364	0.0049
Total			178	55	45 (32)			

Note

Site refers to site codes given in Table 1. Note that diversity statistics for the CLIN (*COI*) and TYGT/GREN populations (*ND1*) are omitted because only one sequence was available.

^a^ Includes multiple sampling sites, see Table 1 and Morrison et al. (2021).

For the *COI* dataset, uncorrected sequence divergence (*D_xy_*) between populations ranged from 0 to 1.69 (Table S1). Sequence divergence (*D_xy_*) among *P. clava* populations averaged 0.55% (range = 0–1.34%), with the highest values involving comparisons with the Maumee drainage (range = 1.08–1.34%). Interestingly, the *P. clava* Maumee populations were more closely related to *P. oviforme* populations from the Cumberland (CUMB), PROC, HOLS, and LTEN populations (*D_xy_* range = 0.32–0.66%) than to more proximate *P. clava* populations. Divergence was low for populations within the Allegheny drainage, including the TYGT (0.11–0.34%), as well as among the Wabash (WABA), Green (GREN), and SCIO populations (0–0.22%). Differentiation was greater among *P. oviforme* populations (average *D_xy_* = 0.88%, range 0.38–1.57%), except for lower divergence between the LTEN and CUMB populations (0.38%). Between‐species comparisons produced a slightly higher average than within *P. oviforme* populations (0.99%), but a range of divergences similar to that observed between *P. oviforme* populations (range = 0.32–1.69). The highest pairwise comparisons involved *P. clava* versus *P. oviforme* from the PROC drainage (*D_xy_* range = 1.43–1.69%).

Similar patterns were observed when net nucleotide differences for the COI dataset were examined (Table S1), with the Maumee drainage producing the highest sequence divergence among *P. clava* populations (*D_a_* range = 0.69–1.05%) yet lower *D_a_* in comparisons with *P. oviforme* from the CUMB, PROC, and HOLS populations (*D_a_* range = −0.09–0.23%). No to minimal net nucleotide substitutions were observed among remaining *P. clava* populations. Greatest *D_a_* distances were observed in comparisons with the PROC drainage (*D_a_* range = 0.90–1.22%, except for the Maumee and CUMB populations mentioned above). Among all collections, genetic differentiation was significant among populations, with *H*s = 0.350 (*p* < .001). Gene flow estimates indicated an *F*_st_ = 0.571 and number of migrants (*N_m_*) = 0.19. No significant deviations from neutrality were detected by Tajima's *D* = −0.936 (*p* > .10) or Fu's *Fs* = −2.310 (*p* > .05).

Uncorrected pairwise percent sequence divergence (*D_xy_*) at *ND1* ranged from 0 (*P. clava* from the GREN and TYGT) to 1.2% (*P. oviforme* from the PROC and Tennessee (TENN); Table S2). Among *P. clava* populations, sequence divergence averaged 0.47% (range = 0.14–0.85%) while comparisons among *P. oviforme* populations were slightly higher, averaging 0.77% (range = 0.34–1.20%). Like the *COI* analyses, the highest divergence estimates among *P. clava* populations involved the Maumee populations (0.45–0.85%). It is noteworthy that the MASJ population was more closely related to the *P. oviforme* Duck (DUCK) and South Chickamauga (SCHK) populations (~0.4%*)* than to other *P. clava* populations (0.52–0.85%). Among *P. oviforme* populations, the PROC/DUCK and the DUCK/SCHK comparisons were lowest (*D_xy_* = 0.34%). Pairwise comparisons between putative species were intermediate, ranging from 0.26% (TENN/TYGT) to 1.05% (TENN/MASJ), and averaging 0.66%. Minimal net nucleotide substitutions (*D_a_*) were detected between several populations, even those involving the two species (e.g., *P. oviforme* from the LTEN, CLIN, and CUMB compared with *P. clava* from the ELK, TYGT, GREN, and SCIO populations). Like the *COI* dataset, genetic differentiation was significant among all collections, with *Hs* = 0.7930 (*p* < .001); however, the fixation index was lower and gene flow estimates were slightly higher for *ND1* (*F*_st_ = 0.2459, *N_m_* = 0.77, respectively). While the *COI* dataset followed expectations under neutrality, the ND1 dataset deviated, with significant values for both Tajima's *D* (−1.964, *p* < .05) and Fu's *Fs* (−34.060, *p* < .001), indicating an excess of rare alleles (Tajima, 1989), suggestive of either a rapid expansion from a population with small effective size (population bottleneck) or positive selection (a selective sweep).

**FIGURE 4:**
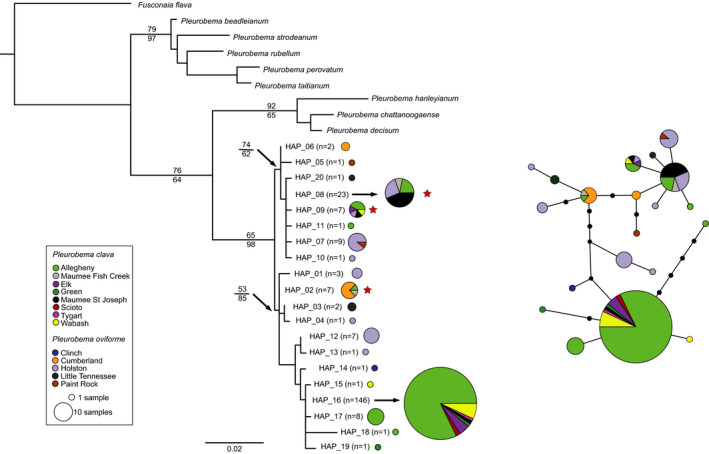
Mitochondrial *COI* (a) maximum‐likelihood (ML) phylogeny (bootstrap support values for ML above and maximum parsimony below branches) and (b) TCS haplotype network for *Pleurobema clava* and *Pleurobema oviforme*. Red stars designate haplotypes present in both species. Colors within pie diagrams indicate populations where given haplotypes were observed

Maximum‐likelihood (ML) and maximum parsimony (MP) phylogenetic analyses included 29 *COI* sequences, of which 20 were the unique *P. clava* and *P. oviforme* haplotypes from this study, plus nine sequences from GenBank representing several closely related *Pleurobema* species (i.e., *P*.* decisum*, *P*. *hanleyanium*, *P*.* beadelianum*, *P*. *rubellum*; Campbell et al., 2005; Inoue et al., 2018; Figure 4a). The *P. clava* and *P. oviforme* sequences formed a single clade that was better supported by MP than ML (support values MP = 98, ML = 65; Figure 4a). Although structuring among *P. clava* and *P. oviforme* haplotypes was weak, two clades were evident, each containing both shared and species‐specific haplotypes. The first clade (support values ML = 74 and MP = 62; Figure 4a) included five *P. oviforme*‐specific (5, 6, 7, 10, and 20) and one *P. clava*‐specific (11) haplotypes as well as two of the three shared haplotypes (8, 9). The second clade (support values ML = 53 and MP = 85; Figure 4a) contained the most common haplotype (16; Figure 4a) that included most of the *P. clava* sequences, plus four additional *P. clava*‐specific (15, 17, 18, and 19) haplotypes, as well as six *P. oviforme*‐specific (1, 3, 4, 12, 13, and 14) and one shared haplotype (2; Table S3).

The two clades recovered in the ML and MP analyses were apparent in the *COI* TCS haplotype network (Figure 4b). It was noteworthy that haplotypes differed by few (1 to 4) substitutions. Shared haplotypes were either intermediate in the network (e.g., Haplotype 2; Figure 4b) or at a distal portion of the network corresponding to Clade 1 in the ML analysis (Figure 4a). Most of the *P. clava* samples had Haplotype 16 (*N* = 146; Figure 4b, Table S3), which was common in the ALLE populations, but was also found in geographically distant populations such as the WABA, MASJ, Maumee Fish Creek (MAFC), ELK, TYGT, and GREN populations. Intermediate haplotypes were comprised mostly of *P. oviforme* sequences from the HOLS and CUMB populations.

**FIGURE 5:**
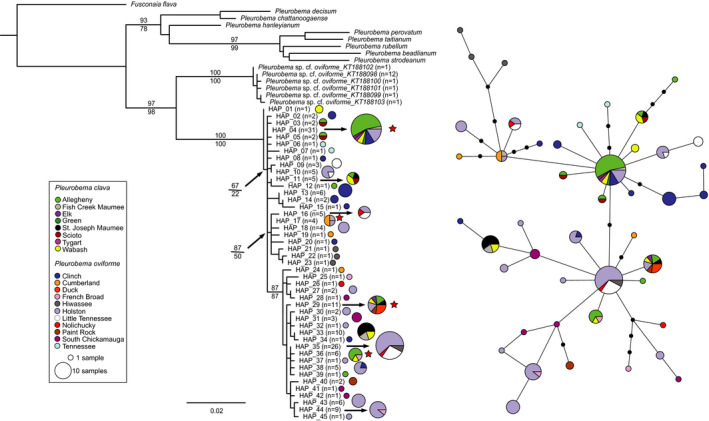
Mitochondrial *ND1* (a) maximum‐likelihood phylogeny (bootstrap support values for ML above and maximum parsimony below branches) and (b) TCS haplotype network for *Pleurobema clava* and *Pleurobema oviforme*. Red stars designate haplotypes present in both species. Colors within pie diagrams indicate populations where given haplotypes were observed

A greater representation of *P. oviforme* was achieved in the *ND1* datasets after the addition of sequence data representing 11 populations from Schilling (2015; Table 1). Maximum‐likelihood and MP phylogenetic analyses included 60 *ND1* sequences, including 45 unique *P. clava* and *P. oviforme* haplotypes, an undescribed but closely related taxon (*P*. cf. *oviforme*, Schilling, 2015), plus several related *Pleurobema* species. As reported in the analysis conducted by Schilling (2015), *P*. cf. *oviforme* was the sister group to *P. clava*/*oviforme* (Figure 5a; uncorrected *p*‐distance = 5.5%, not shown). As in the *COI* phylogenetic analysis, *P. clava*/*oviforme* haplotypes formed a single, well‐supported clade (support values of 100 for both ML and MP; Figure 5a) with minimal differentiation within.

The *ND1* haplotype network incorporating 178 sequences and 45 unique haplotypes formed a star‐like network containing two common central haplotypes with many closely related haplotypes (differing by few substitutions) surrounding the common haplotypes (Figure 5b). Thirty‐four and eight haplotypes were unique to *P. oviforme* and *P. clava*, respectively, while four haplotypes were shared between species (Figure 5b, Table S4), and similarly to observations of the *COI* network (Figure 4b), haplotypes differed by few (1–4) substitutions. The two most common haplotypes (Haplotypes 4 and 35; Figure 5b, Table S4) differed by a single substitution. Haplotype 4 was widely distributed geographically and common in both species, while Haplotype 35 was comprised of *P. oviforme* from the HOLS and LTEN. Three additional shared haplotypes (17, 29, and 36) differed from the most common haplotypes by one to two substitutions. Haplotypes from the HOLS were intermingled throughout the network, along with species‐specific haplotypes.

### 3.2 Morphometrics

**FIGURE 6:**
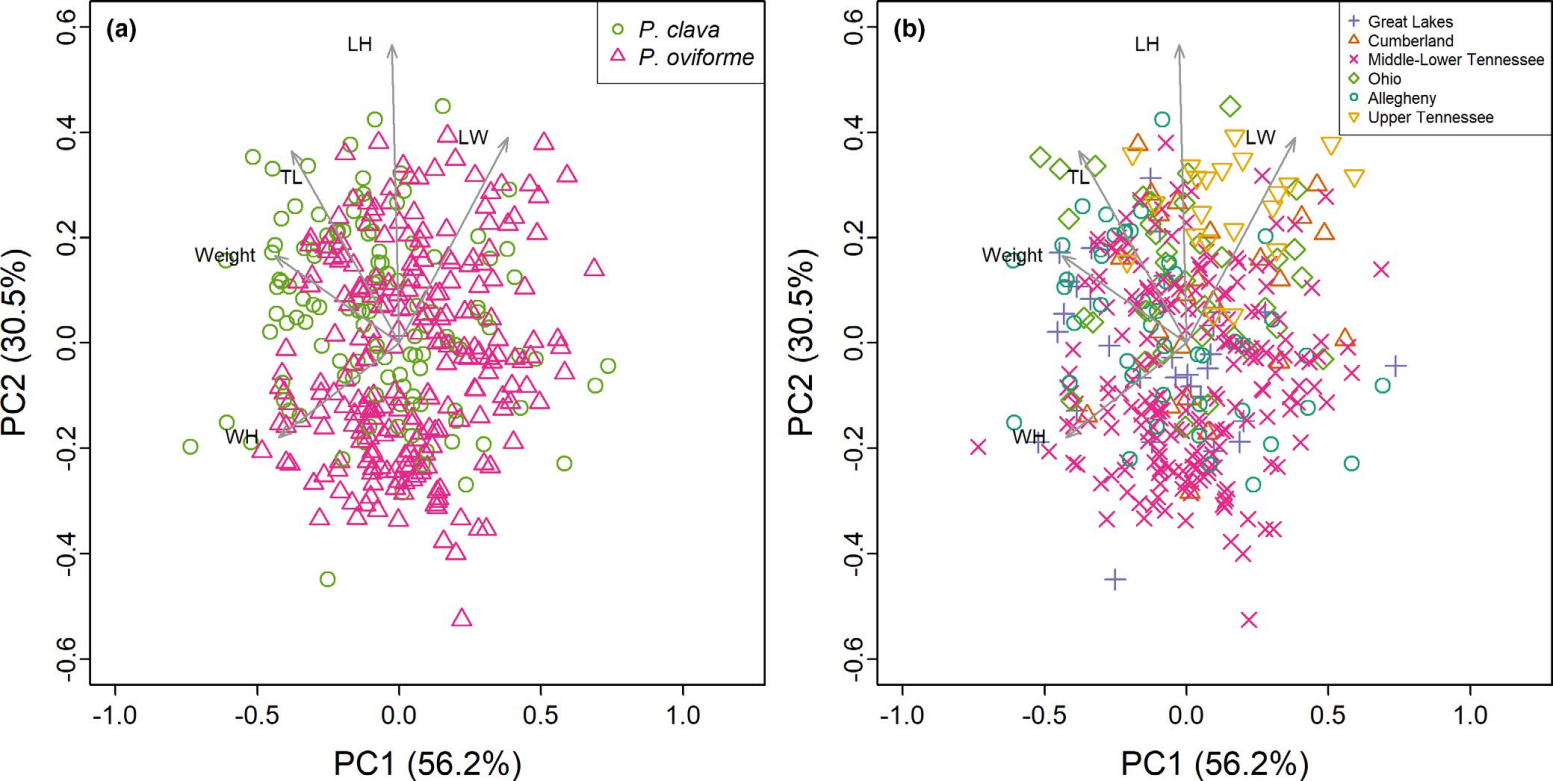
Principal Coordinates Analysis of morphometric data with arrows for log‐transformed biplot variables (WH = width/height; TL = total length; LH = length/height; LW = length/width), with data colored by putative species (a) and by region of collection (b)

Morphometric measurements were taken from 363 museum specimens (*P. clava N* = 129; *P. oviforme N* = 234). The first two PCs described 86.7% of the total variation (Figure 6). The PCA showed broad overlap in morphometric measurements between species (Figure 6a), with all variables besides the length/height ratio loading equally on PC1. PC2 largely described a gradient in length (total length, length/height, length/width). Shells collected from the upper and middle Tennessee drainage were separated on the upper and lower quadrants of the PCA plot (Figure 6b). The PERMANOVA identified significant differences between the nominal species; however, these differences explained a small percentage of the total morphological variation present (*R* ^2^ = 0.030, *F* = 11.344, *p* < .001). Classification to species by the LDA was 58.9% and 72.1% accuracy for *P. clava* and *P. oviforme*, respectively, with an overall classification accuracy of 65.5% (Table 3).

**TABLE 3:** Results of morphological analyses of four shell ratio variables (width to height, width to length, height to length, and weight to length) taken from museum samples of *Pleurobema clava* and *Pleurobema oviforme*

A. Correct Class	*P. clava*	*P. oviforme*	Percent correct classification
*P. clava*	76	53	58.9%
*P. oviforme*	36	93	72.1%
Overall			65.5%
B. Correct Class	*P. clava*	*P. oviforme*	Percent correct classification
*P. clava*	112	23	83.0%
*P. oviforme*	27	136	83.4%
Overall			83.2%

Note

Each confusion matrix shows the number of correctly and incorrectly identified individuals for each species. A. Cross‐validation linear discriminant analysis. B. Random forest analysis.

For the RF analyses, the number of variables tested at each split was set to 2 based on the out‐of‐bag (OOB) error rate estimate. The OOB error rate was 16.8%, indicating that the model had approximately an 83% accuracy for identification of shells to the correct species (Table 3). Based on the mean decrease in the Gini scores, the ratio of shell width to shell height was the most important variable for classification of shells to the correct species, followed by width to length, height to length, and weight to length.

### 3.3 Nuclear microsatellite markers

Of 300 primer pairs tested for amplification, 13 loci were polymorphic and consistently produced alleles within expected size ranges (Table S5). Although several pairwise comparisons for linkage disequilibrium remained significant after correction for multiple tests (adjusted *α* = 0.0006), none were consistent across populations (results not shown). Similarly, only one in 388 tests for departure from HWE was significant (*Pcl278* in the VA_NFH population; Table S6). MICROCHECKER did not detect evidence for large allele dropout or scoring errors; however, null alleles were detected in 10 of 130 locus‐by‐population tests, yet none were consistent across populations. Two MLGs (*Pcla*_PA_AR_HS_02 and *Pcla*_PA_AR_HS_13) differed at only one locus (*PclC211*) and had a kinship value of 0.443. Given the low probability that two unrelated individuals sampled from a single population share the same MLG (*PI* = 4.0 × 10^−14^; *PI*_sibs_ = 2.6 × 10^−6^), this high kinship estimate suggests a possible parent–offspring relationship.

A total of 454 alleles were detected among 235 genotyped samples (Table S5). The number of alleles per locus ranged from 23 (*PclD7*) to 63 (*PclC211*) and averaged 11.5 (Table S6). The mean number of alleles per population ranged from 4.39 (SCIO) to 21 (ALLE Franklin (ALFR), with a mean of 11.5 (Table S6). Following rarefaction to compensate for unequal sample sizes, the WABA and several ALLE populations had the highest allelic richness (5.05–5.15) while the HOLS had the lowest (4.24). Private alleles were detected within all populations, with the MASJ and HOLS populations having most (14 and 17, respectively), while a single private allele was observed in the ELK and GREN populations. Similarly, rarefied private allelic richness estimates were higher for the MASJ and HOLS populations (0.87 and 0.84, respectively), but the TENN and WABA also had high values (0.92 and 0.80, respectively). Observed levels of heterozygosity were generally high, ranging from 0.583 (CUMB) to 0.915 (ALFR), with a global mean of 0.829. Most inbreeding coefficients were negative or close to 0, but two of the *P. oviforme* populations (CUMB and TENN) plus the *P. clava* GREN population had positive inbreeding coefficients, suggesting heterozygote deficits. A likely explanation for these positive inbreeding coefficients is a Wahlund effect given that these samples originated from more than one location within the drainage.

We found little evidence for recent bottlenecks using the microsatellite data from the seven populations with sample sizes of ten or more individuals. No significant results were obtained using the heterozygote excess method (*Hx*) for either the SMM or TPM mutation models (*p* > .00156; Tables S7 and S8), and no significant deviations from a normal L‐shaped distribution were detected (mode shift). Two of the ALLE populations showed significant heterozygote deficits (*H_d_*) under one (ALLE Hunter Station (ALHS)) or both (ALLE West Hickory (ALWH)) mutation models, suggesting that these populations are not in mutation‐drift equilibrium (Table S8).

Several biological hypotheses were assessed using fixation measures (*F*_st_) and AMOVA. First, we tested whether genetic structuring was apparent among all 15 populations. An AMOVA with populations as a second hierarchical level produced a weak but significant global *F*_st_ value of 0.029, with 3% of variation among populations and 97% within populations (Table S9A). We next tested whether greater structuring was apparent between the two putative species, and a significant and greater global *F*_st_ resulted (global *F*_st_ = 0.059, *p *≤ .0001, 4% of variation among putative species; Table S9B). Given that genetic structuring often occurs between watersheds in other unionid species (Galbraith et al., 2015), we also tested whether significant structuring existed between the three watersheds represented in our dataset. Significant structuring was detected, yet the magnitude of differentiation was lower than between species (global *F*_st_ = 0.049, *p *≤ .0001, 3% of variation among watersheds; Table S9C).

Pairwise fixation indices (*G*′_ST_) among *P. clava* populations ranged from 0 (no genetic differentiation, ALLE, MAFC/WABA, and ALLE Walnut Bend (ALWB)/TYGT populations) to 0.056 (moderate genetic differentiation, MASJ vs. SCIO; Table 4) and averaged 0.022 (little genetic differentiation; Hartl & Clark, 1997). All pairwise comparisons with the MASJ population were significant. Similarly, most comparisons involving the WABA population were significant, except for that with the MAFC population. Pairwise *G*′_ST_ values among *P. oviforme* populations ranged from 0.045 to 0.116 (average = 0.075), with two of three comparisons significant (exception CUMB/TENN). Pairwise *G*′_ST_ values between species showed little to moderate differentiation, ranging from 0.013 (MAFC/TENN) to 0.101, HOLS/SCIO; average = 0.053). Pairwise estimates of allelic differentiation (Jost's *D*) showed greater genetic differentiation than fixation measures (*G*′_ST_), indicating that the most common alleles were often not shared between populations. The two measures of differentiation were generally concordant, with low or negative values between populations from the mainstem ALLE, highest estimates in comparisons involving the HOLS population (0.456–0.774), and intermediate estimates involving MASJ and MAFC populations (0.224–0.584), with an exception of the WABA population, which were lower (0.124 and −0.015 between the MASJ and MAFC populations, respectively). Pairwise *D* estimates between *P. clava* populations ranged from slightly negative (ALLE, MAFC/WABA, (ALWB)/TYGT, concordant with *G*′_ST_ estimates) to 0.584 (MASJ/SCIO) and averaged 0.244. Estimates of *D* between *P. oviforme* populations ranged from 0.558 (CUMB/TENN) to 0.774 (CUMB/HOLS) and averaged 0.620. Pairwise *D* values between species ranged from 0.250 (MAFC/TENN) to 0.771 (MASJ/HOLS) and averaged 0.500.

**TABLE 4:** Population pairwise differentiation (fixation index *G*′_ST,_ below diagonal) and Jost's *D* (allelic differentiation, above diagonal) for *Pleurobema clava* and *Pleurobema oviforme*

	1	2	3	4	5	6	7	8	9	10	11	12	13	14	15
	MASJ	MAFC	ALHS	ALWH	ALWB	ALFR	ALFC	ELK	TYGT	WABA	SCIO	GREN	CUMB	TENN	HOLS
	***P. clava***														
P1	‐	**0.304**	**0.390**	**0.333**	**0.326**	**0.366**	**0.410**	**0.430**	**0.234**	**0.124**	**0.584**	**0.464**	**0.320**	**0.481**	**0.771**
2	**0.024**	‐	**0.224**	**0.295**	**0.269**	**0.279**	**0.298**	**0.426**	**0.393**	−0.015	0.513	0.403	0.461	0.250	**0.679**
3	**0.031**	0.017	‐	0.033	−0.036	−0.015	0.035	**0.159**	0.029	**0.244**	**0.362**	**0.325**	**0.443**	**0.572**	**0.614**
4	**0.028**	**0.024**	0.003	‐	−0.007	−0.017	**0.113**	**0.122**	0.039	**0.244**	**0.326**	**0.327**	**0.375**	**0.532**	**0.618**
5	**0.025**	**0.020**	−0.001	−0.003	‐	−0.016	0.089	0.090	−0.044	**0.203**	**0.268**	**0.355**	**0.380**	**0.509**	**0.626**
6	**0.028**	**0.021**	−0.001	−0.001	−0.001	‐	**0.102**	**0.111**	0.015	**0.223**	**0.224**	**0.311**	**0.415**	**0.507**	**0.636**
7	**0.037**	**0.027**	**0.011**	0.003	**0.008**	**0.009**	‐	**0.228**	**0.199**	**0.303**	**0.454**	**0.359**	**0.448**	**0.708**	**0.710**
8	**0.039**	**0.037**	**0.011**	**0.014**	**0.008**	**0.010**	**0.023**	‐	0.080	**0.361**	**0.328**	0.167	**0.423**	**0.593**	**0.645**
9	**0.019**	0.028	0.003	0.002	−0.003	0.001	**0.018**	0.007	‐	**0.236**	**0.405**	**0.384**	0.300	**0.598**	**0.580**
10	**0.010**	−0.001	**0.019**	**0.018**	**0.014**	**0.016**	**0.025**	**0.030**	**0.019**	‐	**0.404**	**0.361**	**0.296**	**0.456**	**0.693**
11	**0.056**	0.045	**0.033**	**0.035**	**0.025**	**0.021**	**0.049**	**0.036**	**0.037**	**0.036**	‐	0.404	0.361	0.296	**0.456**
12	**0.046**	0.035	**0.034**	**0.032**	**0.034**	**0.030**	**0.039**	0.019	**0.036**	**0.033**	0.041	‐	0.331	0.275	**0.643**
13	**0.036**	0.042	**0.043**	**0.048**	**0.041**	**0.045**	**0.053**	**0.049**	0.030	**0.030**	0.040	0.040	‐	0.558	**0.774**
14	**0.036**	0.013	**0.042**	**0.042**	**0.037**	**0.037**	**0.058**	**0.047**	**0.037**	**0.030**	0.029	0.022	0.045	‐	**0.527**
15	**0.093**	**0.082**	**0.076**	**0.073**	**0.074**	**0.075**	**0.093**	**0.085**	**0.071**	**0.079**	**0.101**	**0.092**	**0.116**	**0.064**	‐

Note

Bolded values represent significant pairwise differentiation after 9999 permutations (*p *≤ .05).^a^

^a^ Color coding of cells indicates degree of genetic differentiation after Hartl and Clark (1997): Gray indicates no genetic differentiation (*G*′_st_ =0.00); green indicates little genetic differentiation (*G*′_st_ < 0.05); and yellow indicates moderate genetic differentiation (*G*′_st_ > 0.05–0.15).

**FIGURE 7:**
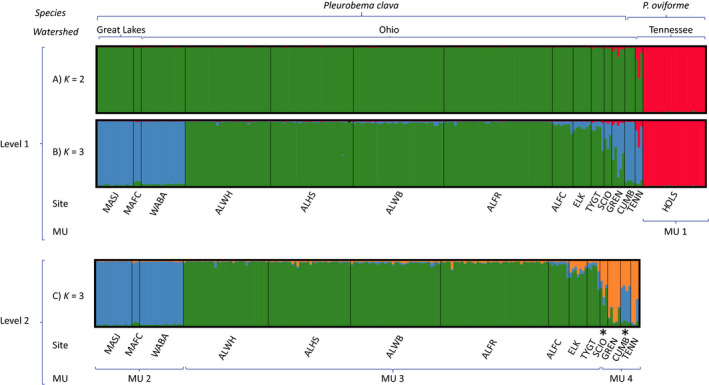
Results from hierarchical STRUCTURE analysis for 15 *Pleurobema* populations. Level 1 of the analysis included all 15 collections with average probability of membership graphs showing individual assignments to either *K* = 2 (a, Δ*K*, Evanno et al., 2005) or *K* = 3 (b, Mean Ln*P*(*X|K*), Pritchard & Wen, 2004) genetic clusters. A second hierarchical analysis included 14 collections and suggested *K* = 3 genetic clusters (c, Level 2). At each level of the analysis, collections were grouped based upon similar Q scores for the next level of analysis. In all graphs, individuals and populations (*SITE*, see Table 1 for population codes) are shown along the *x*‐axis and assignment to populations along the *y*‐axis. Species and watersheds are given at the top and suggested management units (MU) at bottom, with * indicating intermediate Q scores, in which case the collection was included in both possible clusters. No additional structuring was detected within the 3 genetic clusters identified at Level 2

Including 15 populations at the first hierarchical level of analysis, the best‐supported number of clusters detected by STRUCTURE was equivocal. The presence of two clusters (*K* = 2) was suggested as the most likely number of populations by the Δ*K* method (Evanno et al., 2005), in which *P. oviforme* from the HOLS formed a unique genetic cluster while the remaining *P. oviforme* and *P. clava* samples formed a second cluster (Figure 7 Level 1; Figure S1(A)). At *K* = 3, suggested as the most likely number of populations by the mean likelihood of the probability (Mean LnP(X|K)) (Figure 7 Level 1, Figure S1(B)), the HOLS MLGs formed one cluster, the ALLE/ELK/TYGT formed a second cluster, and the MASJ/MAFC/WABA/CUMB/TENN formed a third cluster with the SCIO and GREN populations appearing genetically similar to both the second and third clusters.

The second hierarchical analysis included 14 populations, excluding the unique HOLS population (Cluster or MU1). Both methods suggested *K* = 3 (Figure S1(C–D)), with clusters containing: MASJ, MAFC, and WABA (MU2); the ALLE, TYGT, and ELK (MU3); and GREN and TENN (MU4). Note that the SCIO population appeared admixed between MU3 and MU4, while the CUMB appeared admixed between MU2 and MU4. No additional structuring was detected in subsequent STRUCTURE runs on the three clusters detected at Level 2 (not shown).

Sub‐populations with small sample size tend to merge in STRUCTURE analyses (Puechmaille, 2016), so the analysis was re‐run including five populations with even sample sizes of 17 individuals each (WABA, ALWH, ALHS, ALWB, HOLS). Corroborating results from the full dataset, two genetic clusters were detected at the first hierarchical level, distinguishing the HOLS from other populations (Figure S2(A–C)), and the WABA population formed an independent cluster from the ALLE populations at a second hierarchical level (Figure S2(D–F)). F‐statistics produced by AMOVA were examined to compare the strength of separation between clusters, but not to test for significance since both tests used the same dataset (Meirmans, 2015). The Level 1 *K* = 2 clustering producing the highest *F*_ST_ (0.077; Table S9D) confirming that the HOLS was the most divergent cluster, while *K* = 3 was intermediate (*F*_ST_ = 0.048; Table S9 E) and Level 2 *K* = 3 produced the lowest estimate of fixation (*F*_ST_ = 0.045; Table S9F).

**FIGURE 8:**
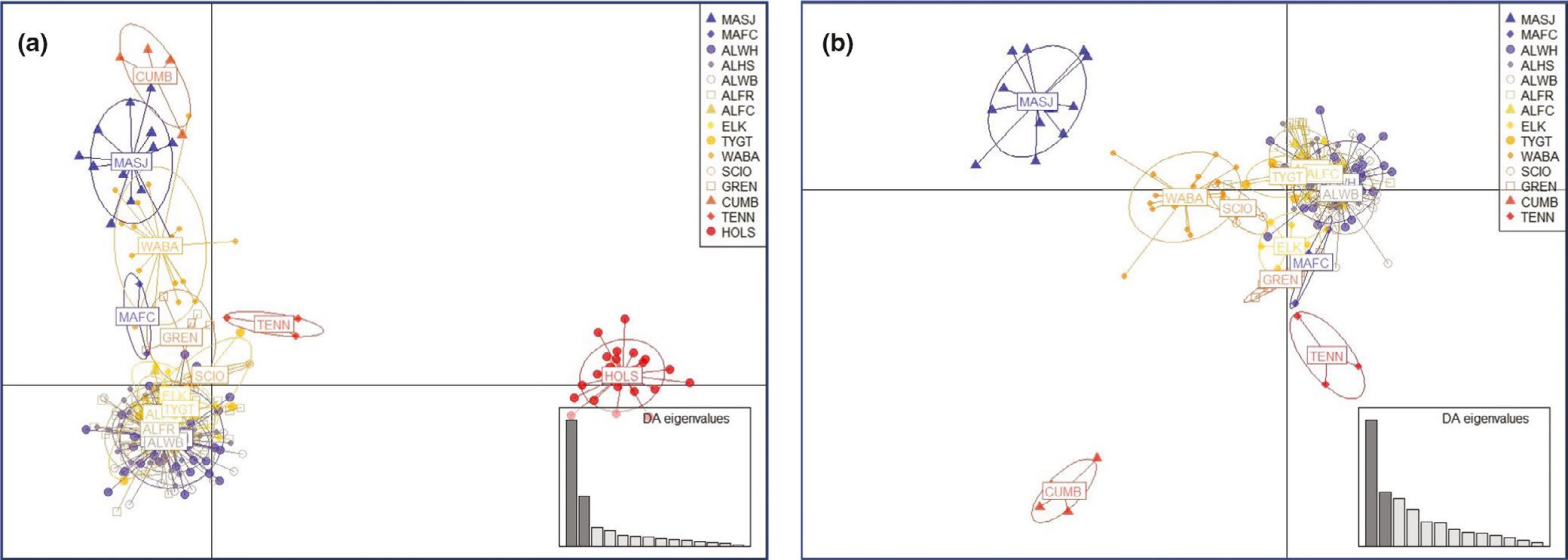
Discriminant Analysis of Principal Components (DAPC) scatterplots based on microsatellite genotype data from *Pleurobema clava* and *Pleurobema oviforme* collections (see Table 1 for collection location and sample sizes). Panel a presents a DAPC scatterplot of 15 collections, where 100 principal components and 14 discriminant function eigenvalues were retained. Panel b presents a DAPC scatterplot of 14 collections, omitting the HOLS collection to better visualize relationships among the remaining collections, and was produced retaining 100 principal components and 13 discriminant function eigenvalues. Note that ALLE, ELK and TYGT populations are not differentiated and are superimposed in the scatterplots

Results of applying the DAPC analysis including all 15 collections (Figure 8a) were consistent with those of STRUCTURE analysis (Figure 7), indicating the *P. oviforme* HOLS was the most differentiated population, while there was substantial overlap between sites from the ALLE, ELK, and TYGT populations. The remaining populations overlapped slightly yet were spread along the *y*‐axis, with the SCIO, GREN, and the MAFC closest to the ALLE group. The placement of the CUMB population of *P. oviforme* suggested genetic similarities with the MASJ and WABA populations, while the TENN *P. oviforme* appeared similar to both the GREN and SCIO *P. clava* populations. A second DAPC analysis was performed on 14 populations after removing the divergent HOLS population (Figure 8b). Again, overlap between sites from the ALLE, ELK, and TYGT populations was apparent, but the CUMB, MASJ, and TENN populations appeared more differentiated. Genetic similarities were suggested between the WABA and SCIO populations, as well as the ELK/GREN/MAFC populations.

To investigate evolutionary relationships among allele frequencies, pairwise Nei's *D_A_* distances were illustrated as a mid‐point rooted neighbor‐joining dendrogram (Figure S3). Most population groupings were concordant with those from the STRUCTURE analyses (Figure 7). The clade including the MASJ/MAFC/WABA populations corresponds to MU2 in the STRUCTURE graph (Figure 7c), and the clade including ALLE plus the ELK/TYGT populations corresponds to MU3 (Figure 7c). Also concordant with the STRUCTURE analysis, the SCIO and CUMB populations were intermediate between the MU groupings and basal to MU2 and MU3.

A Mantel test revealed a significant correlation between geographic river distances and genetic distance (Nei's *D*, *r* = .5851, *p* = .004, Figure S4), suggesting a stepping‐stone model of dispersal (Kimura & Weiss, 1964).

## 4 DISCUSSION

Given the long history of taxonomic uncertainty surrounding *P. clava* and *P. oviforme*, the purpose of our study was to test the taxonomic status and infer the population genetic structure of these two nominal species. Specifically, we used sequences of two mtDNA genes and morphometrics analysis of external shell measurements to test the hypotheses that these two taxa are either separate species with parapatric distributions or a single widely distributed species throughout the Ohio River, Tennessee River, and lower Great Lakes watersheds. Additionally, to help guide future conservation and management efforts, we utilized data on variation at 13 DNA microsatellite loci to characterize population genetic structure under a null hypothesis that populations represent a single readily interbreeding, undifferentiated unit with shared adaptations and a common evolutionary trajectory (Vignieri et al., 2006). While synonymy of *P. clava* and *P. oviforme* has been suspected for nearly a century based on similar shell morphologies (Ortmann, 1925), only recently have molecular data been applied to address species boundaries within the clubshell species complex, where phylogenetics and species delimitation analyses using *COI* sequence data suggested that *P. clava* and *P. oviforme* were conspecific (Campbell & Lydeard, 2012; Campbell et al., 2005, 2008; Inoue et al., 2018). Here, we have increased sampling of both putative species and coverage of geographic ranges, as well as evaluated relationships using morphometrics and multiple mtDNA genes (*COI* and *ND1*), providing the additional geographic and genetic sampling suggested by Inoue et al. (2018). A holistic approach using both molecular and morphological data sampled across the range of the taxa of interest is needed to increase understanding of often complex relationships and to better inform conservation strategies (Jones et al., 2006; Jost et al., 2018; Pante et al., 2015). Whether these taxa represent two species or a single species with multiple MUs has important implications for their future ESA listing status and conservation management. The genetic markers and the geographic coverage of the DNA sample collections utilized in this study have analytical strengths and weaknesses and underlying methodological assumptions affecting data interpretation. Hence, we discuss the strengths and weaknesses of our study results, emphasizing the caveats and key inferences from each evidence type (i.e., geography, mtDNA, nuclear DNA, morphology) and how they may affect delineation of species, MUs, and our population genetic and phylogeographic understanding of these two species.

### 4.1 Mitochondrial DNA evidence for a single species

Evidence for *P. clava* and *P. oviforme* being a single species was apparent in both the *COI* and *ND1* mitochondrial datasets. First, phylogenetic analysis of each mtDNA gene revealed monophyletic relationships with minimal differentiation among intermingled haplotypes (Figures 4 and 5), providing evidence for a single species based upon the PSC. Additionally, haplotype sharing between clubshell taxa was detected at both mtDNA genes, blurring any obvious genetic lines that may be drawn between the putative species. Several common haplotypes were widespread geographically, crossing nominal species and watershed boundaries. Generally, levels of mtDNA sequence divergence observed between clubshell populations were less than interspecific divergence observed in previous studies of unionid mussels (Boyer et al., 2011; Burdick & White, 2007; Inoue et al., 2014; Johnson et al., 2018; King et al., 1999; Pfeiffer et al., 2016; Pieri et al., 2018; Roe & Lydeard, 1998; Smith et al., 2019). Sequence divergence was less than 2% in all pairwise population comparisons at mtDNA genes and was moderate between putative species (0.99 and 0.66% for *COI* and *ND1*, respectively; Tables S3 and S4), with shared haplotypes between *P. clava* and *P. oviforme* at both genes, resulting in no clear “barcode gap” (Meyer & Paulay, 2005) that would be expected between intra‐ and interspecific divergence comparisons. There were also cases where the intraspecific genetic variation exceeded interspecific divergence. Examples include higher divergence between the Maumee and other *P. clava* populations compared with the CUMB, HOLS, and PROC *P. oviforme* populations (*COI*; Table S1) and versus the DUCK and SCHK *P. oviforme* populations (*ND1*; Table S2).

A caveat regarding our conclusion of a single clubshell species is the imbalance in population sampling among datasets, with our *COI* dataset favoring collections of *P. clava* from the Ohio River basin, while more balanced sampling throughout the ranges of both putative species was achieved at *ND1*, especially from the HOLS and CLIN drainages. Given the broader sampling of populations in the CUMB, HOLS, and TENN drainages at the *ND1* locus, this dataset provides the most compelling evidence for a single clubshell species; however, more balanced sampling at all genetic markers would strengthen results. It is noteworthy that an undescribed taxon from the Little River clustered separately in our phylogenetic analysis of *ND1* data (Figure 5; Schilling, 2015). This result highlights the utility of this gene region in delineating cryptic taxa when they are present in a dataset, and shows potential for undocumented cryptic diversity within the clubshell species complex requiring additional taxonomic investigation.

While numerous studies of freshwater mussels have utilized mtDNA to assess relationships at various evolutionary depths, this locus reflects only one component of evolutionary history. Low information content and potential non‐neutrality (i.e., selective sweeps and introgression) bring into question sole reliance on the mtDNA locus to define evolutionary lineages (Ballard & Whitlock, 2004; Edwards & Beerli, 2000; Galtier et al., 2009; Moritz, 1994; Rubinoff & Holland, 2005). Discordance between mtDNA gene trees and species trees may be expected in recently diverged lineages, as ancestral polymorphisms may be retained, a process that may be more pronounced in structured populations (Arbogast et al., 2002; Avise, 2000; Maddison, 1997; Wakeley, 2000, 2001). The significant neutrality tests at *ND1* may be indicative of selective sweeps and retention of ancestral polymorphisms, although both selection and historical demography (e.g., population bottlenecks) must be taken into consideration (Nielsen, 2005). For clubshells, bottlenecks were not detected at microsatellite loci (Table S5), making selective sweeps the more likely scenario, therefore drawing into question the reliance on these results to discern species.

By increasing the number of loci and through joint analysis of gene trees, greater success in delimiting closely related lineages has been achieved in other studies (Dupuis et al., 2012; Fujita et al., 2012; Herrera & Shank, 2016). Additionally, well‐developed mtDNA sequence divergence is not always evident even among distinguishable species. For example, Jones et al. (2006) showed that several species of *Epioblasma* occurring in the Tennessee and Cumberland River basins are diverged morphologically but are minimally diverged (<1%) at several mtDNA genes and even share some of the same haplotypes among the respective populations. There are numerous examples in the scientific literature of closely related species that are distinct morphologically, behaviorally, and ecologically, but are minimally diverged at mtDNA and even share haplotypes, including some mollusks, freshwater and marine fishes, birds, and other taxa (Avise, 2000; Echelle & Dowling, 1992; McCartney et al., 2013; Pedraza‐Marrón et al., 2019). Clearly, the addition of morphological characters and nuclear loci strengthens our findings.

### 4.2 Morphological differences between the shell forms

Quantitatively assessing the morphological variation between *P. clava* and *P. oviforme* is challenging due to the overall similarity of their shell shape, the inherent variation in the shell morphology throughout the ranges of both species, and the potential taxonomic confusion that has occurred in the parapatric regions of each species' distribution. The taxonomic challenges are exacerbated by the over‐description of morphological variation in this species complex, which has resulted in at least 16 and 23 described species that are now considered synonyms of *P. clava* and *P. oviforme*, respectively (Graf & Cummings, 2021; Watters et al., 2009; Williams et al., 2008). Additionally, the type specimen of *Unio oviformis* is apparently lost (Graf & Cummings, 2021; Williams et al., 2008) and the type specimen of *Unio clava* is a relatively large specimen (75 mm total length) with severe growth scars on the shell disk, which is not considered a diagnostic character for the species (Lamarck, 1819; Watters et al., 2009; Williams et al., 2008).

In the early 1900s, A. E. Ortmann was very aware of the morphological similarity between *P. clava* and *P. oviforme*, even stating that they could be the same species (i.e., synonymous), but he was also aware of the considerable variation observed in the headwater versus big‐river forms of *P. oviforme* in the Tennessee River system (Ortmann, 1918, 1920, 1924, 1925). He recognized the inflated big‐river form as *P. oviforme* “*holstonense*,” which he observed primarily in the lower and middle Tennessee River and the lower reaches of its tributaries, including the Duck and Paint Rock rivers, and he recognized the compressed headwater form as *P. oviforme* “*argenteum*,” which he observed mostly in the upper Tennessee River and its tributaries. But he also collected the compressed “*argenteum*” form from the upper reaches of the Duck, Elk, and Paint Rock rivers and other smaller streams, indicating that this form also was known from the lower and middle regions of the Tennessee River system, but seemingly restricted to the headwater areas of the tributaries (Ortmann, 1920, 1924). Further, the inflated “*holstonense*” form was known from the upper Tennessee River basin, but mainly from the mainstem of the Tennessee River and the very lower reaches of the Clinch, Holston, French Broad, and Nolichucky rivers (Ortmann, 1918). Hence, some level of clinal variation in the shell morphology of *P. oviforme* from headwater to larger‐river environments throughout the Tennessee River basin is well established in the historical literature and in museum records for the species. While this clinal shell variation likely occurs in the Cumberland River system as well, *P. oviforme* is primarily known from headwater areas where shell inflation differences are less pronounced. Regardless, the overall shape of what has been nominally identified as *P. oviforme* from the Cumberland River system is quite similar to that of *P. clava*. In fact, individuals collected from the lower to middle Cumberland River system occasionally have been identified as *P. clava*, contributing to confusion about each species' distribution in this river system. The observed morphological variation is likely a product of ecophenotypic plasticity, driven by the diverse geological conditions of the respective watersheds, with the predominately dolomite‐limestone based Valley and Ridge province of the Powell, Clinch, and Holston valleys, the sandstone‐shale strata of the Cumberland Plateau, and the igneous‐metamorphic strata of the Blue‐Ridge mountains drained by the Nolichucky, French Broad, Little Tennessee, and Hiwassee river valleys. In the upper Tennessee River basin, specimens identified as *P. oviforme* generally have a more rounded and flattened shape, especially in the headwaters of the major tributaries to this region. Thus, excluding the parapatric regions of each species' distribution in the lower and middle Tennessee River and Cumberland River, the shell forms exhibited by most populations in the upper Tennessee River basin are rare in the Ohio River and Great Lakes region that is generally understood to be the distribution of *P. clava*; however, the lack of a type specimen of *U. oviformis* creates difficulty assigning a species name to the shell forms commonly observed in the Tennessee and Cumberland river systems.

We measured commonly employed morphological measurements (shell length, height, width, and weight) and the respective ratios as response variables to delineate these taxa over a broad geographic range. While results of the PERMANOVA suggested significant differences in shell shape, broad overlap in shell shapes across species was apparent in the PCA and only 3% of the variance in shell shape was explained, implying a high degree of morphological overlap between the two nominal taxa (Figure 6). However, for taxa that are morphologically very similar, a limited set of measurements may not be sufficient to discriminate them. For example, it is likely that PCA analyses utilizing these same variables to delineate numerous “pigtoe” taxa in the genera *Fusconaia*, *Pleurobema*, and *Pleuronaia* would show only minor differences and therefore a high degree of morphological overlap in shell shape. Examples of highly similar shell morphologies for species in the Tennessee River system include the following: *Fusconaia cor* and *F*. *cuneolus*; *Pleurobema cordatum* and *Pleurobema plenum*; and *Pleuronaia barnesiana* and *Pleurobema oviforme*. All of these taxa are well diverged genetically and have other morphological traits used to distinguish among them (Hyde et al., 2020; Schilling, 2015); therefore, similarities in shell phenotype among these taxa are likely due in part to phenotypic plasticity.

Using LDA and Random Forest analyses, our overall classification rates between the two species were 65.5% and 83.2%, respectively (Table 3). Some of the misclassifications may in part be due to the morphological similarity (e.g., width‐to‐length ratio) of *P. oviforme* from the Duck and Paint Rock river samples to those of *P. clava*. Additionally, we do not report herein morphological differences or classification rates among the various populations that we examined. For example, the Random Forest analysis classification rate for individuals from the North Fork Holston River is >90%. Beyett et al. (2020) showed that traditional measurements and ratio‐based methods were not as precise in assigning species to genetic groups or species. Conversely, Inoue et al. (2014) showed that shell width may be very important in differentiating among species and that 2D shape (using geometric approaches) was less important. A combination of methods that use multiple landmark measurements spread uniformly across the shell and accounting for shell width may prove useful to further characterize differences between populations in the clubshell complex (Hyde et al., 2020; Schilling, 2015; Willsie et al., 2020). Our morphological analyses assessed differences between the taxa using samples collected throughout the broad geographic range of each species, and our morphometric analysis is congruent with Ortmann’s (1925) assessment indicating that the morphologies of *P. clava* and *P. oviforme* may represent clinal variation of a single species.

### 4.3 Nuclear DNA population divergence and structure

Many previous studies of unionids have detected low levels of mtDNA divergence, with minimal divergence among haplotypes sampled across geographic ranges, while microsatellites provided increased resolution of population structuring (Chong et al., 2016; Jones et al., 2006; Kelly & Rhymer, 2005; Scott et al., 2020; Zanatta & Murphy, 2006, 2007). Similarly, analyses of nuclear microsatellite markers identified low to very high levels of among‐population structuring among clubshell populations. The STRUCTURE analysis (Figure 7) suggested two to four unique genetic clusters that may be considered MUs following the criteria of Moritz (1994) given the significant differences in frequencies of nuclear alleles. The most genetically unique cluster included the HOLS population in Virginia. Concordantly, the largest transition in the distribution of genetic variation in the microsatellite dataset separated the HOLS population from others (Table 4). The HOLS population had moderate pairwise *G*′_st_ values (0.071–0.116), but very high pairwise *D*_est_ estimates (0.456–0.774; Table 4), formed a unique cluster at all tested *K* values (Figure 7), and clearly separated from other populations in the DAPC analysis (Figure 8a). These divergence values exceeded known values observed between other *Pleurobema* species; for example, Jones, Johnson, et al. (2015), utilizing similar DNA microsatellite loci, reported *D* values of 0.24 between sympatric populations of *P*. *cordatum* and *P*. *plenum* in the Green River, KY, and 0.42 between allopatric populations of *P*. *cordatum* in the Green River, KY, and *P*. *plenum* in the Clinch River, TN. A second genetic cluster included the ALLE, TYGT, and ELK clubshell populations of the upper Ohio Basin. A close genetic relationship among these populations also was supported in the DAPC (Figure 8) and neighbor‐joining (Figure S3) analyses. The third genetic cluster included the two Maumee populations (MASJ and MAFC) as well as the Tippecanoe (WABA), which was supported by the DAPC (Figure 8) and neighbor‐joining (Figure S3) analyses. A potential fourth cluster included the GREN and LTEN populations (Figure 7c), although the genetic similarity of these populations is suggestive but less clear in the DAPC (Figure 8) and neighbor‐joining (Figure S3) analyses, and sample sizes were low. Therefore, four genetic clusters or MUs (Figure 7) are the best‐supported and biologically relevant grouping based on our limited geographic sampling of *P. oviforme*, where the SCIO and GREN populations showed admixture with the second and third clusters, while the CUMB and TENN showed admixture with the third (Maumee and WABA) cluster.

A significant IBD pattern (Figure S4) indicates a strong correlation between genetic divergence and increasing geographic distance, supporting a stepping‐stone model of gene flow (Kimura & Weiss, 1964) rather than dispersed and divergent. Isolation‐by‐distance patterns have been supported within several unionid species in North America (Berg et al., 1998, 2007; Elderkin et al., 2008; Ferguson et al., 2013; Inoue et al., 2013; Kelly & Rhymer, 2005; Rowe & Zanatta, 2015) and Europe (Zieritz et al., 2010); thus, the stepping‐stone model of gene flow may often be appropriate for stream‐dwelling organisms with clumped distributions (Elderkin et al., 2008). The microsatellite markers also demonstrated finer‐scale divergence, such as that between populations in the mainstem ALLE River and the French Creek tributary (Table 4).

Significant caveats regarding our analysis of population structuring should be recognized. First, increased sample representation of known populations within the CUMB and HOLS drainages as well as the Clinch River population, which was not analyzed using microsatellites in this study, would likely refine our knowledge of barriers to gene flow and allow greater precision in defining the geographic extent of MUs. Small sample sizes from some populations coupled with highly polymorphic loci could lead to biased estimates of genetic distances (Ruzzante, 1998) and genetic clustering (Puechmaille, 2016). Similarly, while the nuclear microsatellites adequately clustered populations, refinement of these clusters and their geographic boundaries (and suggested MUs) may be feasible through increased investigation of the nuclear genome utilizing high‐throughput sequencing approaches such as Restriction Site Associated DNA sequencing (RADseq; Baird et al., 2008; Garrison et al., 2021). Considering that underlying genetic structuring may be linked to adaptive differentiation, utilizing transcriptomic single nucleotide polymorphism (SNP) data may be informative as well.

While it would be ideal to have a balanced set of samples for all datasets, the combination of molecular markers discussed here provides historical insight into the evolution of these lineages. Our analysis is strengthened by this multi‐locus approach with representation from both the nuclear and mitochondrial genomes. In the future, increasing sampling and genotyping of the CUMB and HOLS populations and including assessment of the robust Clinch River population and other populations would be desirable to inform management actions. At least a dozen populations of *P*. *ovifome* in the Tennessee River basin have yet to be sampled and analyzed at nuclear DNA loci, which would greatly advance our understanding of genetic diversity and levels of population differentiation within and between the two species.

### 4.4 Geologic history and phylogeographic structure

Advance and retreat of glaciers during and after the Pleistocene had dramatic effects on topography, stream characteristics, and temperature regimes that likely influenced genetic diversity and structuring among North American freshwater populations, including fishes (Bernatchez & Wilson, 1998; Morrison et al., 2006; Near et al., 2001; Strange & Burr, 1997) and unionids (Elderkin et al., 2007, 2008; Hewitt et al., 2019; Inoue et al., 2013, 2015; Jones et al., 2015; Pieri et al., 2018; Scott et al., 2020; Zanatta & Harris, 2013). Although several studies of unionids have found that structuring generally follows major hydrogeologic basins (Galbraith et al., 2015; Mock et al., 2010), our STRUCTURE results for *P. clava* and *P. oviforme* do not align fully with present‐day drainages. Although several geological events could explain genetic connections that do not align with present drainages (e.g., tectonic activity, general erosional stream capture events), one possible scenario involves ancient drainage connections that occurred as ice sheets covering the northern portion of the range retracted approximately 15,000–8,000 years ago (Dyke & Prest, 1987). For example, the close genetic relationship between the Maumee and Wabash drainages seen in shared mitochondrial haplotypes and an MLG‐based genetic cluster (MU2; Figure 7) may be the result of shared connections over the past 13,600 years. Historically, the St. Joseph River drained into the Wabash (Bleuer & Moore, 1972) and was part of an “Interior Highlands” group of drainages (Mayden, 1988). More recently, this connection was cut off and the St. Joseph River was captured by the Maumee drainage (Graf, 2002; Pielou, 1991), creating a potential route for colonization to the Great Lakes watershed. Several fishes, including a known host for *P. clava* (Northern Hogsucker, *Hypentelium nigricans*, Berendzen et al., 2003), and unionids (Threeridge *Amblema plicata*, Elderkin et al., 2007; Flutedshell *Lasmigona costata*, Hewitt et al., 2016; Plain Pocketbook *Lampsilis cardium*, Hewitt et al., 2019) also show genetic evidence for a colonization pathway into the Great Lakes region via the Wabash‐Maumee outlet, the western portion of which is now the Wabash drainage. Genetic similarities between other drainages also reflect ancient connections. For example, MU3 (including the ALLE, TYGT, and ELK populations; Figure 7) were included in the ancient “Upper Ohio,” while MU4 (GREN and LTEN populations) comprised populations from the “Lower Ohio” (Mayden, 1988). The two populations that appear admixed (SCIO and CUMB) lie in the center of the geographic distribution and the detected admixture may be a result of former versus present‐day stream connections.

Several studies of connectivity and colonization history among unionid populations in eastern North America have suggested multiple refugia from glaciation (Beaver et al., 2019; Hewitt et al., 2019; Inoue et al., 2013). Two glacial refugia are suggested from the data presented here, including the ancient Cumberland and Tennessee Rivers. The Cumberland shows genetic connections with the Maumee and Wabash drainages (Figures 7, 8, and S3), while the Tennessee River was basal to all populations (Figure S3). Despite low divergence among populations, the *ND1* dataset provided evidence regarding colonization history. Results of a test of neutrality of the *ND1* dataset suggested a rapid expansion from a once‐small effective population size. The combination of low nucleotide diversity, high haplotype diversity (since there are many haplotypes with few mutations between them), and a star‐shaped haplotype network (Figure 5) suggests the signature of recent demographic expansion consistent with expansion from glacial refugia. Further, more than 90% of the *ND1* haplotypes occurred only in the UTRB, suggesting that this geographic region harbors a high amount of unique genetic variation, suggesting long‐term persistence and accumulation of genetic diversity. Maintaining this adaptive capacity will be critical for any conservation efforts targeting *P. oviforme* because the Clinch River in the UTRB contains one of the few remaining demographically robust populations for this species (Fitzgerald et al., 2021).

## 5 MANAGEMENT IMPLICATIONS AND CONCLUSIONS

Our mtDNA, nuclear DNA, and morphological datasets represent the largest to date aimed at assessing the relatedness among *P. clava* and *P. oviforme* populations. Although some discrepancies exist among the datasets, each enriches our interpretation of the complex evolutionary history underlying present‐day patterns of similarities and divergence among populations (Bowen et al., 2005; Butlin et al., 2008).

Three significant findings are evident from our analyses. First, strong mtDNA divergence that is typical among well‐diverged mussel species was lacking, suggesting that *P. clava* and *P. oviforme* represent a single species. Given the genetic similarity and sharing of mtDNA haplotypes across populations and the observed admixture of individuals at nuclear DNA microsatellites in key geographically intermediate populations, we consider the presence of a single species the most likely biological scenario. Second, our morphometric analyses showed overlap in shell shape and lacked congruence with current models of two species. Instead, morphological differences were more pronounced among certain population‐level comparisons relative to species‐level comparisons, suggesting that phenotypic differences in shell shape may be the result of ecophenotypic plasticity and not genetic differentiation. The shells, for example, from both the HOLS and CLIN populations are essentially indistinguishable morphologically but are quite distinguishable from shells of *P. clava* in the Ohio River and Great Lakes watersheds because they tend to be more compressed and rounded with umbos heavily eroded and weakly elevated with respect to the hinge. This morphology is distinctive from the original description of *U. oviformis*, where the umbos of the species were described as “prominent, not decorticated.” Third, analyses of the DNA microsatellite data suggest that significant structuring exists across the broad geographic ranges of *P. clava* and *P. oviforme*, providing critical information for defining representative units to inform ESA decision making and recovery efforts. Four genetic clusters, or MUs, were detected through STRUCTURE analysis, only some of which follow present‐day drainages. The most distinctive genetic cluster was comprised of the HOLS population, which ideally should be investigated further by increasing sampling and microsatellite genotyping of geographically proximate populations, such as those from the Clinch River and its tributaries, which likely comprise the largest population of the species range‐wide. If CLIN mussels are closely related to HOLS, these two populations and perhaps others in the UTRB (e.g., French Broad (FRBR), Hiwassee (HIWA), Little River, Nolichucky (NOLI), PROC, SCHK) would form at a minimum a large MU. Alternatively, sampling intermediate populations may erode genetic clusters through the inclusion of genetically admixed individuals, which has been shown in groups characterized by strong patterns of isolation by distance (Mason et al., 2020).

Our data will aid management efforts to assess how the synonymy of *P. clava* and *P. oviforme* may impact extinction risk assessments and can be used to inform strategies for translocating individuals and augmenting populations. Because *P. oviforme* is currently being considered for ESA protection and *P. clava* is listed as federally endangered, our findings suggesting that these are the same species have direct implications for upcoming ESA decisions. Synonymizing these species may remove the need to determine an individual status for *P. oviforme* and require that the current status and recovery strategies for *P. clava* be re‐evaluated based on the combined distributions. However, if future genetic, morphological, life history, and breeding studies demonstrate that *P. clava* and *P. oviforme* or some of the geographically isolated populations are different species from each other, then synonymizing these two taxa now could have major negative consequences for their protection and management. Based upon genetic structuring and diversity of multiple unionid species, Galbraith et al. (2015) proposed translocating and augmenting populations within watersheds as a prudent management strategy. However, the four MUs detected in our analyses do not group strictly by watershed, but more closely align with ancient (Pleistocene) river connections. Hence, until additional genetic and morphological data are available to inform species boundaries between *P. clava* and *P. oviforme*, and reproductive data are available to inform breeding outcomes and the fitness of progeny produced from broodstock out‐crossings, we suggest that population management could occur and be restricted to the MU level. Finally, high levels of genetic diversity were observed using both nuclear microsatellites as well as mtDNA haplotypes in most populations. Since both abundance and genetic diversity are important when considering the status of imperiled populations, high diversity is a positive sign for long‐term stability and potential to adapt to changing environments. Similarly, little to no evidence of population bottlenecks suggests that population sizes were stable historically. Therefore, there may not be an immediate need to augment populations solely for the purpose of enhancing genetic diversity. Along with the increased sampling at nuclear loci for *P. oviforme* populations suggested above to further define the boundaries among evolutionary lineages, host‐fish compatibility trials and additional research regarding ecological and demographic characteristics may guide decisions regarding taxonomy.

## CONFLICT OF INTEREST

None declared.

## AUTHOR CONTRIBUTIONS

**Cheryl L. Morrison:** Conceptualization (equal); Data curation (lead); Formal analysis (equal); Funding acquisition (lead); Investigation (equal); Methodology (equal); Resources (equal); Visualization (equal); Writing‐original draft (lead); Writing‐review & editing (lead). **Nathan A. Johnson:** Conceptualization (equal); Data curation (supporting); Formal analysis (equal); Funding acquisition (supporting); Investigation (equal); Methodology (equal); Resources (equal); Visualization (equal); Writing‐original draft (supporting); Writing‐review & editing (equal). **Jess W. Jones:** Conceptualization (equal); Data curation (supporting); Formal analysis (equal); Funding acquisition (supporting); Investigation (equal); Methodology (equal); Resources (equal); Visualization (equal); Writing‐original draft (supporting); Writing‐review & editing (supporting). **Michael S. Eackles:** Conceptualization (supporting); Data curation (supporting); Formal analysis (supporting); Investigation (equal); Methodology (supporting); Writing‐original draft (supporting); Writing‐review & editing (supporting). **Aaron W. Aunins:** Formal analysis (supporting); Visualization (supporting); Writing‐original draft (supporting); Writing‐review & editing (supporting). **Daniel B. Fitzgerald:** Formal analysis (supporting); Methodology (supporting); Visualization (supporting); Writing‐original draft (supporting); Writing‐review & editing (supporting). **Eric M. Hallerman:** Funding acquisition (supporting); Investigation (supporting); Methodology (supporting); Resources (supporting); Visualization (supporting); Writing‐review & editing (supporting). **Tim L. King:** Conceptualization (equal); Funding acquisition (supporting); Investigation (supporting); Methodology (equal); Resources (equal).

## Supporting information

Figure S1‐S4

Table S1‐S9

## Data Availability

DNA sequences: GenBank accessions COI MT991776–MT991982; ND1: MW005982–MW006051. Sampling locations, DNA sequence alignments, morphological data, and microsatellite genotypes are available in Morrison et al., 2021 at USGS ScienceBase https://doi.org/10.5066/P928BVHR.
