# Supplementary material for: Genetic and morphological characterization of the freshwater mussel clubshell species complex (Pleurobema clava and Pleurobema oviforme) to inform conservation planning
Source: Ecol Evol. 2021 Oct 20;11(21):15325–50. doi: 10.1002/ece3.8219 (PMC8571583; doi:10.1002/ece3.8219)
Supplement: Supplementary file 1 — Figure S1‐S4 [file ECE3-11-15325-s001.pdf]

Supplemental Figures for:

Genetic and morphological characterization of the clubshell species complex (*Pleurobema clava* and *P. oviforme*) to inform conservation planning

Cheryl L Morrison*[1], Nathan A Johnson[2], Jess W Jones[3], Michael S Eackles[1], Aaron W Aunins[1], Daniel B Fitzgerald[1], Eric M Hallerman[4], Tim L King[1]

[1]U.S. Geological Survey, Eastern Ecological Science Center, Leetown Research Laboratory, Kearneysville, WV 25430, USA

[2]U.S. Geological Survey, Wetland and Aquatic Research Center, Gainesville, FL 32653, USA

[3]U.S. Fish and Wildlife Service, Department of Fish and Wildlife Conservation, Virginia Polytechnic Institute and State University, Blacksburg, VA 24061-0321, USA

[4]Department of Fish and Wildlife Conservation, Virginia Polytechnic Institute and State University, Blacksburg, VA 24061-0321, USA

*Corresponding Author: cmorrison@usgs.gov

List of Supplemental Figures

S1 Plots of Mean LnP(*K*) and Delta *K* used to interpret the number of genetic clusters from hierarchical STRUCTURE analyses from genotypes of 13 microsatellite loci in *P. clava* and *P. oviforme* populations shown in Figure 7 of the main text. Plots A and B resulted from analysis of all 15 populations at the first hierarchical level, while plots C and D resulted from 14 populations at the second level.

S2 Plots of Mean LnP(*K*) and Delta *K* used to interpret the number of genetic clusters from hierarchical STRUCTURE analyses from genotypes of 13 microsatellite loci in: (A-B) four *P. clava* and one *P. oviforme* populations with even samples sizes (N=17), with resulting STRUCTURE graph (C); and (D-E) four *P. clava* with even samples sizes with resulting STRUCTURE graph (F).

S3 Mid-point rooted neighbor-joining tree generated from pairwise Nei's D_A genetic distances from 13 microsatellite loci showing relationships among *Pleurobema clava* and *P. oviforme* populations (see Table 1 for population codes). Suggested Management Units (MUs) refer to the STRUCTURE analysis, Figure 7. * indicates intermediate Q scores in STRUCTURE results.

S4 Plot comparing pairwise genetic distances among *Pleurobema clava* and *P. oviforme* populations (Nei's D_A,) with geographic river distances (km). The density of points was estimated using the Kde2d package in R. The image function in R was used to visualize these densities through the use of a color palette, where red is the highest density. yellow is intermediate, and blue is low density. The relatively even distribution of points along the regression line suggests a continuous gradient in IBD across the sites examined.

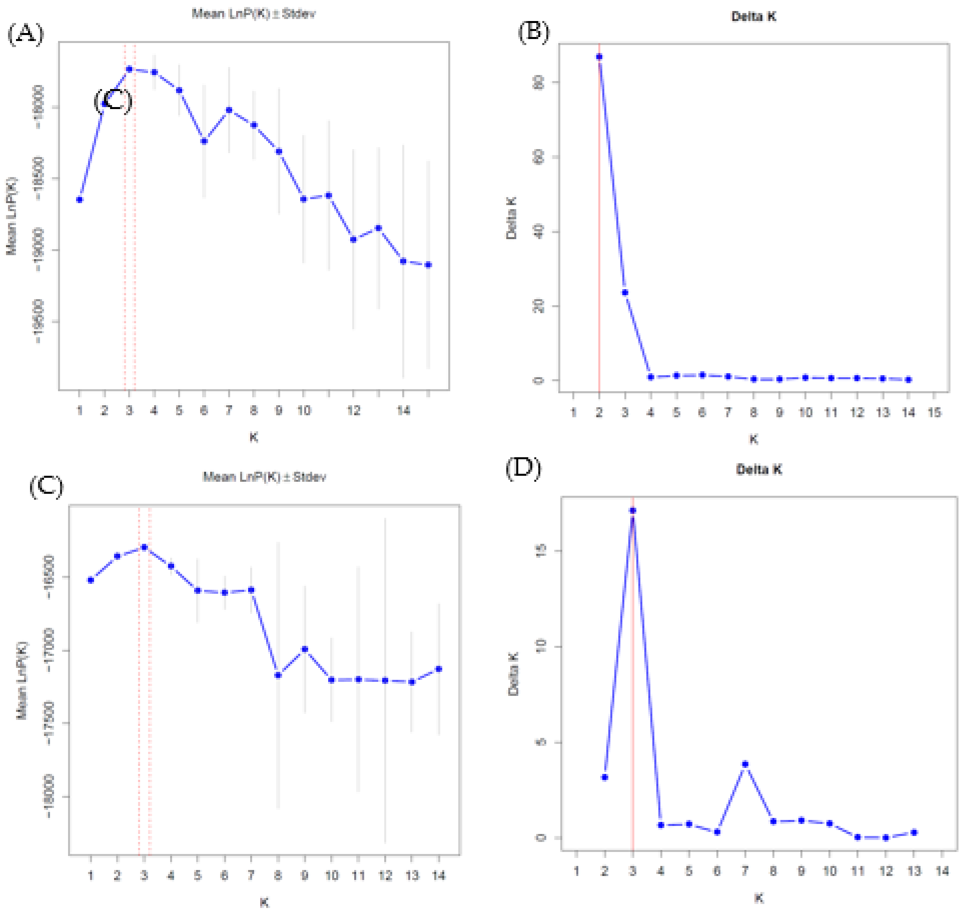

Figure S1: Plots of Mean LnP(*K*) and Delta *K* used to interpret the number of genetic clusters from hierarchical STRUCTURE analyses from genotypes of 13 microsatellite loci in *P. clava* and *P. oviforme* populations shown in Figure 7 of the main text. Plots A and B resulted from analysis of all 15 populations at the first hierarchical level, while plots C and D resulted from 14 populations at the second level.

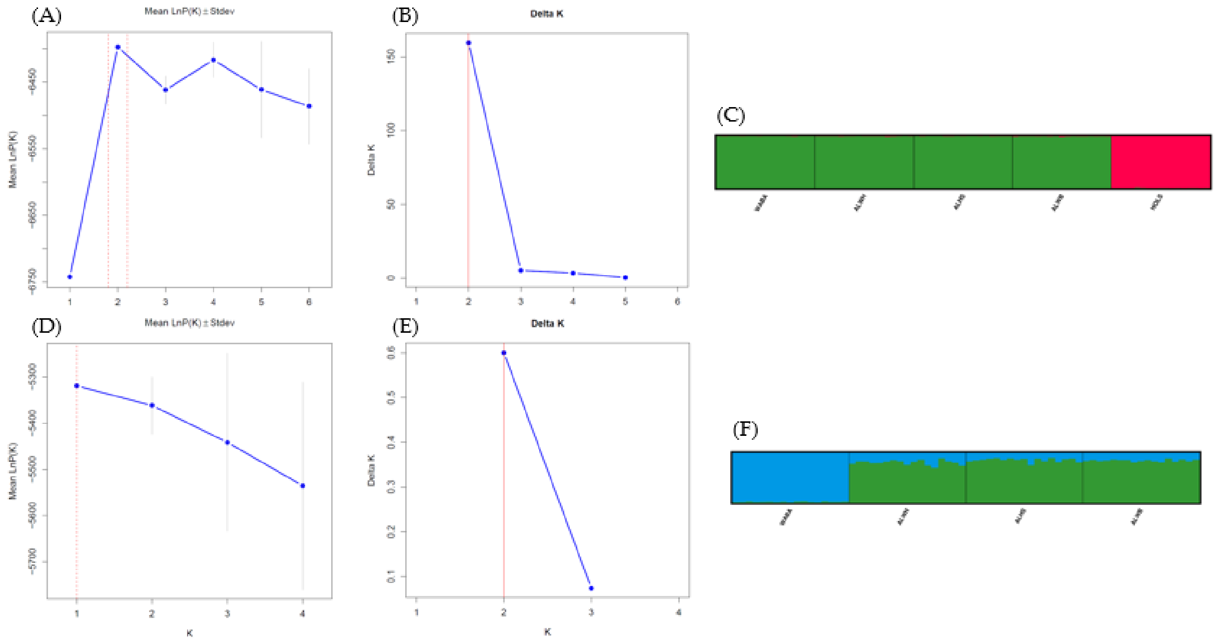

Figure S2: Plots of Mean LnP(*K*) and Delta *K* used to interpret the number of genetic clusters from hierarchical STRUCTURE analyses from genotypes of 13 microsatellite loci in: (A-B) four *P. clava* and one *P. oviforme* populations with even samples sizes (N=17), with resulting STRUCTURE graph (C); and (D-E) four *P. clava* with even samples sizes with resulting STRUCTURE graph (F).

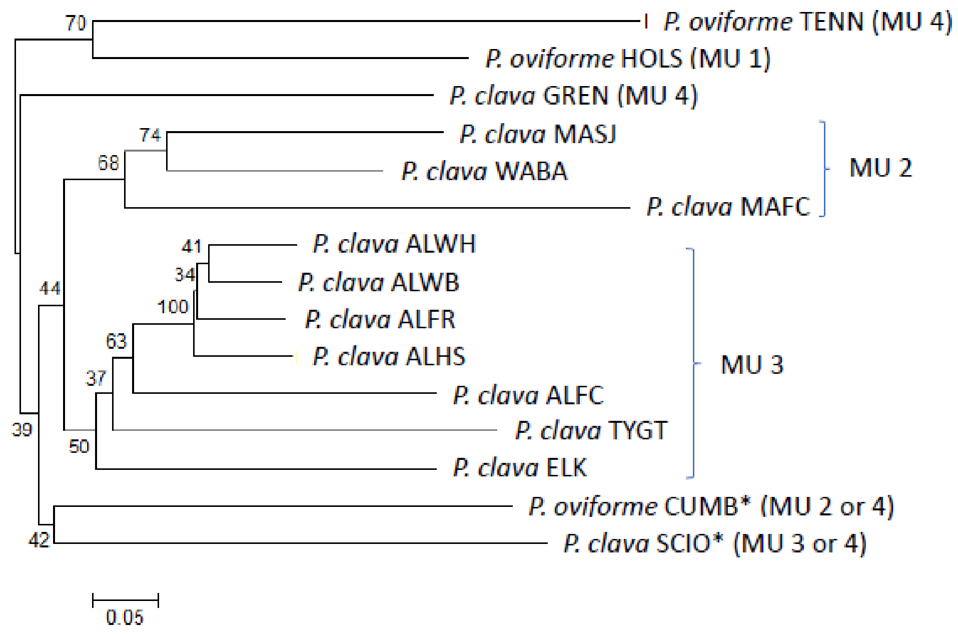

Figure S3: Mid-point rooted neighbor-joining tree generated from pairwise Nei's DA genetic distances from 13 microsatellite loci showing relationships among *Pleurobema clava* and *P. oviforme* populations (see Table 1 for population codes). Suggested Management Units (MUs) refer to the STRUCTURE analysis, Figure 7. * indicates intermediate Q scores in STRUCTURE results.

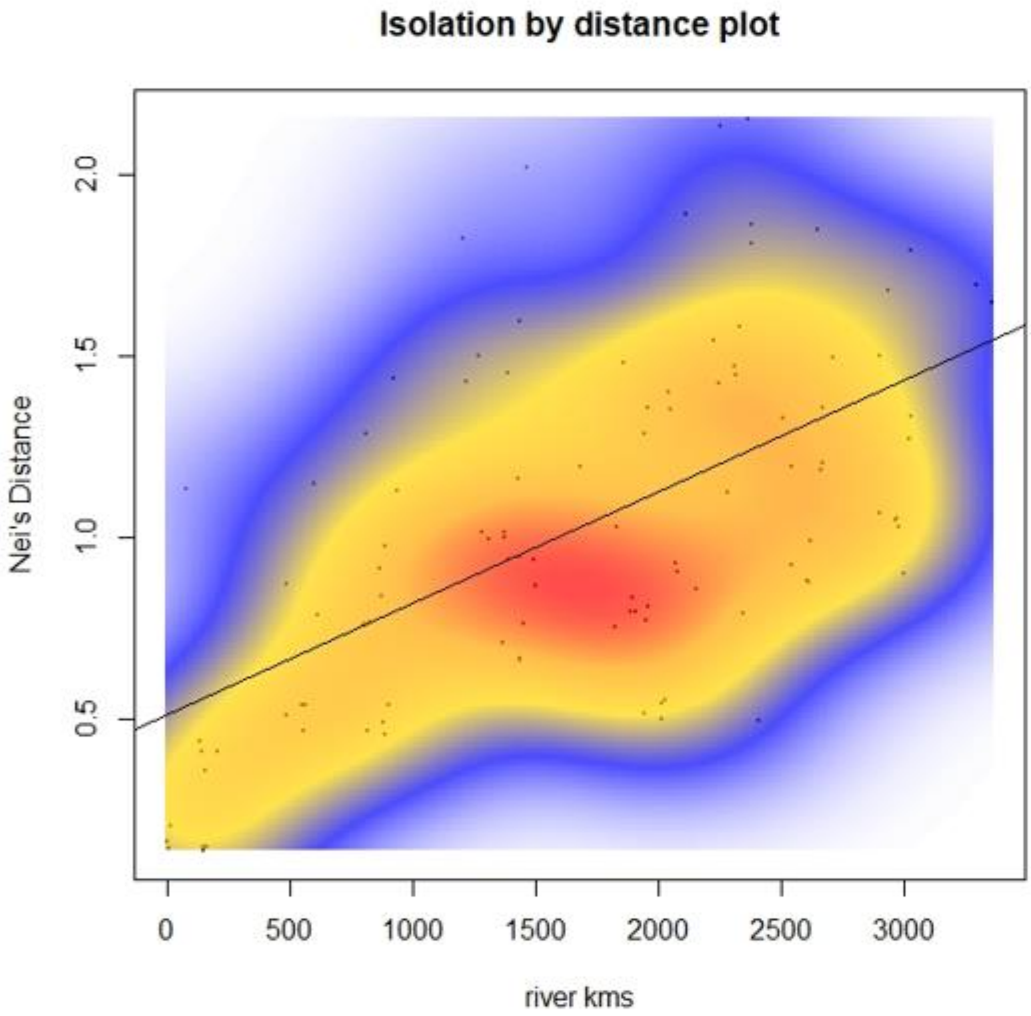

Figure S4: Plot comparing pairwise genetic distances among *Pleurobema clava* and *P. oviforme* populations (Nei's D_A,) with geographic river distances (km). The density of points was estimated using the Kde2d package in R. The image function in R was used to visualize these densities using a color palette, where red is the highest density. yellow is intermediate, and blue is low density. The relatively even distribution of points along the regression line suggests a continuous gradient in IBD across the sites examined.
